# The “Bear” Essentials: Actualistic Research on *Ursus arctos arctos* in the Spanish Pyrenees and Its Implications for Paleontology and Archaeology

**DOI:** 10.1371/journal.pone.0102457

**Published:** 2014-07-16

**Authors:** Maite Arilla, Jordi Rosell, Ruth Blasco, Manuel Domínguez-Rodrigo, Travis Rayne Pickering

**Affiliations:** 1 Àrea de Prehistòria, Universitat Rovira i Virgili (URV), Tarragona, Spain; 2 IPHES, Institut Català de Palaeoecologia Humana i Evolució Social, Tarragona, Spain; 3 The Gibraltar Museum, Gibraltar, Gibraltar; 4 IDEA (Instituto de Evolución en África), Museo de los Orígenes, Madrid, Spain; 5 Department of Prehistory, Complutense University, Madrid, Spain; 6 Department of Anthropology, University of Wisconsin-Madison, Madison, Wisconsin, United States of America; 7 Evolutionary Studies Institute, University of the Witwatersrand, Johannesburg, South Africa; 8 Plio-Pleistocene Palaeontology Section, Department of Vertebrates, Ditsong National Museum of Natural History (Transvaal Museum), Pretoria, South Africa; University of Oxford, United Kingdom

## Abstract

Neotaphonomic studies of large carnivores are used to create models in order to explain the formation of terrestrial vertebrate fossil faunas. The research reported here adds to the growing body of knowledge on the taphonomic consequences of large carnivore behavior in temperate habitats and has important implications for paleontology and archaeology. Using photo- and videotrap data, we were able to describe the consumption of 17 ungulate carcasses by wild brown bears (*Ursus arctos arctos*) ranging the Spanish Pyrenees. Further, we analyzed the taphonomic impact of these feeding bouts on the bones recovered from those carcasses. The general sequence of consumption that we charted starts with separation of a carcass’s trunk; viscera are generally eaten first, followed by musculature of the humerus and femur. Long limb bones are not broken open for marrow extraction. Bears did not transport carcasses or carcass parts from points of feeding and did not disperse bones appreciably (if at all) from their anatomical positions. The general pattern of damage that resulted from bear feeding includes fracturing, peeling, crenulation, tooth pitting and scoring of axial and girdle elements and furrowing of the upper long limb bones. As predicted from observational data, the taphonomic consequences of bear feeding resemble those of other non-durophagus carnivores, such as felids, and are distinct from those of durophagus carnivores, such as hyenids. Our results have paleontological and archaeological relevance. Specifically, they may prove useful in building analogical models for interpreting the formation of fossil faunas for which bears are suspected bone accumulators and/or modifiers. More generally, our comparative statistical analyses draw precise quantitative distinctions between bone damage patterns imparted respectively by durophagus (modelled here primarily by spotted hyenas [*Crocuta crocuta*] and wolves [*Canis lupus*]) and non-durophagus (modelled here by brown bears and lions [*Panthera leo*]) carnivorans.

## Introduction

A major focus of neotaphonomic research is to understand the cause-and-effect relationships that are involved when modern carnivores act as bone-modifying agents. From paleontological and archaeological perspectives, a major objective of this kind of research is to use what we learn about the taphonomic impact of carnivores in order to, in stepwise fashion, isolate evidence of non-carnivore, presumably hominin-induced effects in fossil fauna palimpsests. It is from that point that hypotheses of carcass foraging by ancient human ancestors can proceed.

Much of previous paleontologically and archaeologically directed neotaphonomic research has concentrated on durophagus carnivorans, including especially hyenids (e.g., [1]–[7]). But there are exceptions to this generalization, which include important work on bone-modifying felids (e.g., [8]–[15]), birds (e.g., [16]–[18]), crocodiles (e.g., [19]–[20]) and even bacteria (e.g., [21]), among other taxa. Ursids have also been occasional taphonomic subjects of paleontologists and archaeologists. Here we add to this last body of research, presenting the results of our study of the taphonomy of ungulate carcasses consumed and modified by free-ranging brown bears (*Ursus arctos arctos*) in the Spanish Pyrenees.

Fossils of the genus *Ursus* (including the extinct cave bear species *Ursus deningeri* and *Ursus spelaeus*) are very common in assemblages from European Pleistocene sites–including those that also contain hominin remains and hominin-produced stone artifacts. These regular paleontological associations of cave bear fossils and especially Neanderthal (*Homo neanderthalensis*) fossil and archaeological remains formed the basis of dramatic and influential hypotheses that Neandertals both hunted and worshipped cave bears (e.g., [22]–[27]).

In contrast to their prominent role in scenarios of ancient European life, from a neotaphonomic perspective, ursids are among the least studied of all common, large carnivores. Given that most experts recognize that bears were very likely significant taphonomic agents in past (especially in karst contexts, where their hibernation-related activities and probable cannibalistic inclinations held great potential to modify the spatial distribution and condition of the bones of their conspecifics and other animals (e.g., [28]–[40]), this imbalance is doubly unfortunate.

We acknowledge that *Ursus arctos arctos* is obviously not an exact match for its prehistoric congeners (e.g., [41]–[43]). For instance, unlike Pleistocene bears, extant Pyrenean brown bears face little to no feeding competition for large carcasses (see below) and, depending on their exact distribution on the landscape, have access to different arrays and quantities of other foods. These differences probably impact the relative intensity of bear feeding behaviors, as well as condition their decision making processes about whether to transport (or not transport) carcass parts to sheltered feeding sites. We do not believe, however, that these differences disqualify modern brown bears as suitable taphonomic referents for extinct cave bears. Instead, we highlight relevant continuities between the two types of bear: modern brown bears are about the same size as were cave bears [41]; the skulls of both taxa have “domed” frontals and prominent, similarly placed sites for the attachments of large temporalis and masseter muscles [44]–[46], imbuing both with powerful bite forces [43], [45]; both–based on behavioral observations for modern brown bears and on isotopic [37], [47], [48] and occlusal microwear [49] evidence for cave bears–are/were omnivorous.

Couturier’s [50] work in the Cantabrian Mountains (northern Spain) was the first study to describe the sequence by which modern brown bears consumed large vertebrate carcasses, but his observations were also unsystematic and lacked a contemporary taphonomic focus. It was almost thirty years later that Haynes [51]–[53] became the first researcher to describe modifications of selected skeletal elements of cattle (*Bos taurus*) fed to captive bears; Haynes then used these results to interpret taphonomic damage on carcass remains of wild North American ungulates. Haynes’s observations and conclusions–that bears usually abandon feeding on a carcass once it has been defleshed and eviscerated, and that this behavior produces only minimal taphonomic damage, including minor tooth pitting, scoring, furrowing, crushing and perforating of cortical bone (see below)–have often been recruited in analogical models that assert ursid involvement in the accumulation and/or modification of some Pleistocene fossil faunas (e.g., [54], [32], [33], [36], [37]).

More recently, Saladié et al. [55] fed fleshed, but disarticulated, limb segments of young cattle, pigs (*Sus scrofa*) and sheep (*Ovis aries*) to captive brown bears in the Barcelona Zoo and in the Hosquillo Natural Park (Spain). In contrast to the general patterns of bear-induced taphonomic damage documented by Haynes [51]–[53], Saladié et al. [55] found that bears generated bone surface destruction and modification patterns very comparable to those produced by other, better-studied large carnivores. Such patterns include: a high degree of bone breakage; the abundant production of “diaphyseal cylinders” (i.e., long limb bone [LLB] specimens that lack epiphyses, which were chewed-off, but that retain intact, full-circumferenced shafts); the furrowing, and scooping-out of trabeculae from LLB epiphyses. Saladié et al. [55] also showed that the metric range of individual bear-produced tooth marks overlaps with that previously documented for African lions (*Panthera leo*).

Confusing these apparently clear taphonomic patterns were the conclusions of Sala and Arsuaga [56], who tracked the modification of nine domestic equid carcasses consumed by free-ranging brown bears in the Cantabrian Mountains. In addition to the interesting behavioral data that Sala and Arsuaga [56] phototrap study yielded (e.g., the Cantabrian bears often moved carcasses before feeding on them, but never into caves or other secluded refuges; observations that are obviously relevant to models that prehistoric bears collected carcasses and bony residues in shelter sites), their taphonomic results, in contrast to those of Saladié et al. [55], included much reduced intensities of tooth-marking and fracturing of bones and clustering of most tooth marks on axial, rather than on appendicular, skeletal elements. Sensibly, Sala and Arsuaga [56] attribute the stark difference in the taphonomic patterns that emerged from their study and those documented by Saladié et al. [55] to the fact that the former was conducted with free-ranging subjects and whole carcasses and the latter with captive animals and selected carcass portions of much smaller ungulates.

In summary, each study in the development of research on brown bears as taphonomic agents has been an incremental improvement to its predecessor(s); each has also proven of great value in the interpretation of fossil faunas suspected to have been (at least partially) formed and/or modified by prehistoric bears. By presenting new data and conclusions on the taphonomic behaviors and consequences of free-ranging brown bears in the Pyrenees of Lleida (Spain), here we contribute to the evolving research program on this critically important but understudied large carnivore. Importantly, our study advances the state of knowledge about brown bears as taphonomic agents because our study group is composed of free-ranging bears who were granted unfettered access to complete, fresh carcasses of seventeen ungulates.

In addition, we contextualize our results by presenting a cross-species, multivariant analysis, comparing our taphonomic data to those generated in other studies on other major large carnivore taphonomic agents, including spotted hyenas (*Crocuta crocuta*), lions and wolves (*Canis lupus*). (Taphonomic data on brown hyenas [*Parahyaena brunnea*] and striped hyenas [*Hyaena hyaena*] are utilized more restrictively in selected analyses.) In doing so, this analysis achieves a broader goal of further refining those taphonomic signatures that are respectively diagnostic of durophagus (modelled here by spotted hyenas and wolves) and non-durophagus (modelled here by brown bears and lions) carnivorans. These results will prove useful to paleontologists and archaeologists investigating questions about Pleistocene large mammal terrestrial ecosystems.

## Materials and Methods

### Ethics Statement

All the research images analysed in this study were obtained by the monitoring teams from the Conselh Generau d’Aran and DAAM (Departament d’Agricultura, Ramaderia, Pesca, Alimentació i Medi Natural) of the Generalitat de Catalunya and funded by the Ministerio de Agricultura, Alimentación y Medio Ambiente from Spanish Government. All the carcasses were recovered under the supervision of these teams, who are the responsible of the protection of wildlife in the studied areas (Pallars Sobirà and Valh d’Aran, Spain), and managed the administrative permissions required, including the owner’s permission in the case of domestic animals. No animals were damaged or sacrificed by the researchers and/or rangers during the present project.

### Study area and subject population

**Figure 1:**
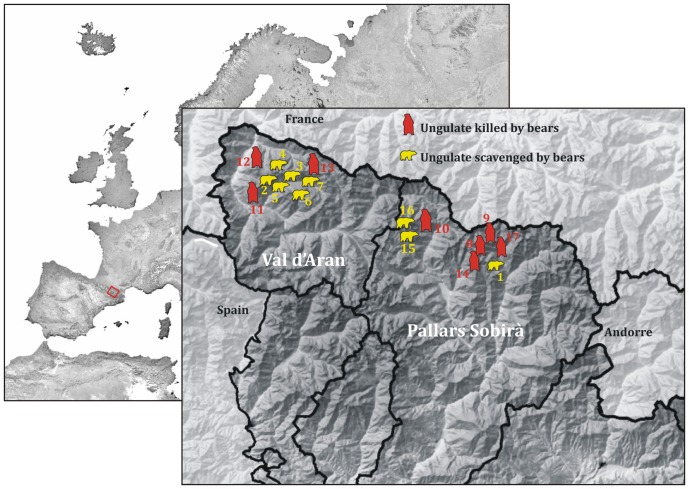
The Spanish Pyrenees showing the location of the recovered carcasses.

The Pyrenees mountain system extends westward from the Mediterranean Sea to the Atlantic Ocean, separating the Iberian Peninsula from the rest of Europe (Figure 1). The formation of the range, related to the Alpine orogeny, resulted in metamorphic peaks (some of which attain altitudes of 3,000 m above sea level), with deep valleys in between. The region is characterized by an Alpine climate and concordantly graded vegetation, dependent on relative altitude. The Pyrenees are sparsely populated by humans, who subsist there largely on a mixed economy, based mostly on animal husbandry and tourism.

Brown bears were naturally distributed in the Pyrenees until the recent historical period, but were all but extirpated by the 1950s. Transporting wild brown bears from Eastern Europe (mostly Slovenia), a reintroduction program, under the banner of *Project Life* (funded by the European Union and the French, Catalonian and Aragonian governments), began in 1996. Since that time, the brown bear population has been continuously monitored (originally with external satellite-connected transmitters and other intraperitoneal devices and now by use of photo- and videotraps, direct observation, hair traps and fecal collection), as has the transformations of their range. Today, the Pyrenean brown bears comprise a controlled population of 25–30 individuals, with 12–14 adults of a balanced sex ratio.

In order to assure the bears’ recurrence there, current photo- and videotrap locales are baited with fruits, ungulate carcasses and glandular odors. It is at these locales that research/monitoring teams are able to regularly observe the bears’ health and behavior. Based on longitudinal data generated at observation points, adult males have annual ranges of 700–2,000 km^2^, while females have annual ranges of <300 km^2^. Individuals of both sexes range between 1,600–2,300 m above sea level, across the intramountain regions of Pallars Jussà and Pallars Sobirà (in the Mediterranean watershed) and Val d’Aran (in the Atlantic watershed). Bear diet in these regions is primarily herbivorous but is supplemental by some animal product gained through predation and scavenging of ungulate (including wild red deer [*Cervus elaphus*], fallow deer [*Dama dama*] and roe deer [*Capreolus capreolus*], as well as domestic cattle, sheep, goats [*Capra hircus*] and horses [*Equus caballus*]) and smaller vertebrate carcasses, as well as some insectivory. Because of the absence of other large carnivores in the monitored regions, bears face a distinct lack of interspecific competition for edible resources from vertebrate carcasses. This factor probably accounts, at least in part, for the observation that bears generally feed recurrently on a single large carcass for anywhere from only one to just less than eight weeks.

### Bear-derived sample

We analyzed the remains of seventeen variously sized ungulate carcasses fed on by bears in Pallars Sobirà and Val d’Aran during spring and summer months between 2010 and 2013 (Figure 1). For this sample, Table 1 summarizes the number and identity (age, sex, weight) of bear consumer(s) per carcass, carcass taxon, estimated ontogenetic age of carcass and state of carcass completeness when collected by our research team. These carcasses were made available to the bears by: (1) bear predation; (2) bear scavenging of natural deaths; (3) road kills transported by forest rangers to observation points along routes used by bears. We were able to document bear consumption of carcasses in the case of predation on domestic animals because livestock owners notified bear monitoring teams of those kills. In cases of bear scavenging, monitoring teams installed photo- and videotraps in the vicinity of carcasses. Thus, human intervention with carcasses was minimal until cessation of bear feeding, which was signaled by prolonged lack of visits by bears to the carcass. Exact location and spatial dispersion of the remains of each carcass were documented using a GPS and camera.

**Table 1:** General characteristics of bears and ungulate carcasses they consumed, organized by individual observations (OB).

Observation	Bear*			Other carnivores present	Consumed carcass				
	Age in years/sex	Weight (kg)	Season		Carcass	Characteristics	Cause of death	Dispersion	Date of death
OB 1	5/M	80	Summer 2010		Horse	Adult M	Natural	Articulated	July 2010
OB 2	Unknown bear	Unknown	Spring 2011	*Vulpes, Martes*	Roe deer	Adult M	Unknown	Articulated	April/May 2011
OB 3	Unknown bear	Unknown	End of spring 2011	*Vulpes, Martes*	Roe deer	Adult	Unknown	Articulated	June 2011
OB 4	24/M	230–270	Spring 2010		Red deer	Juvenile F	Roadkill	50 m^2^	April 2010
OB 5	24/M	230–270	End of spring 2011	*Vulpes, Martes*	Roe deer	Juvenile	Unknown	100 m^2^	June 2011
OB 6	Unknown bear	Unknown	Unknown	*Vulpes, Martes*	Red deer	Adult	Natural	70 m^2^	Unknown
OB 7	16/F	160–170	End of summer 2010	*Vulpes, Martes*	Cow	Adult	Natural	250 m^2^	September 2010
OB 8	Unknown bear	Unknown	Summer 2012		Sheep	Adult/Senile	Bear predation	Articulated	July 2012
OB 9	Unknown bear	Unknown	Summer 2012		Sheep	Adult	Bear predation	Articulated	July 2012
OB 10	12/F (+offspring)	140	Summer 2012		Sheep	Juvenile/Adult	Bear predation	Articulated	August 2012
OB 11	Unknown bear	Unknown	Summer 2012		Sheep	Infant	Bear predation	Articulated	August 2012
OB 12	Unknown bear	Unknown	Summer 2012		Goat	Juvenile	Bear predation	Articulated	July/August2012
OB 13	Unknown bear	Unknown	Summer 2012		Sheep	Adult	Bear predation	Articulated	July/August2012
OB 14	Unknown bear	Unknown	End of summer 2012		Sheep	Senile	Bear predation	Articulated	September 2012
OB 15	2/M	30–50	Spring 2013		Red deer	Juvenile M	Roadkill	Semi-articulated (<4 m^2^)	May 2013
OB 16	Unknown bear	Unknown	Spring 2013		Red deer	Juvenile	Roadkill	Articulated (<2 m^2^)	May 2013
OB 17	Unknown bear	Unknown	Summer 2013		Sheep	Adult	Bear predation	100 m^2^	July 2013

*In some cases, we registered the specific identification of bear/s involved in this experiment from traits such as skin blemishes, control-earrings or necklaces. In the cases of unknown bears, the speed of action (bear predation-attacks or natural death of prey) and/or display problems prevented the identification of the specific bear/s involved in the OBs. Abbreviations: M = Male; F =  Female.

### Taphonomic analyses

Based on body masses of living adult animals, the sample of carcass remains was divided into three categories: large (>300 kg; includes cattle and horses), medium (300–100 kg; includes red deer), and small (<100 kg, includes roe deer, sheep and goats). Similarly, the bear consumers were divided into large (>100 kg) and small (<100 kg) individuals.

We used strong incandescent light and 10x power hand lens, as well as an Olympus SZ 11 stereoscopic (magnification up to 110x), together with an ESEM (FEI QUANTA 600 Environmental Scanning Electron Microscope), to identify and classify bone surface damage on the collected ungulate bones, following established standards (e.g., [51], [57]–[60]). The damage we quantified included:

1. *Tooth pits* and *punctures* are caused by the penetration of the tooth (or teeth) of taphonomic agent into (pitting) or through (puncturing) a bone’s cortex. Both types of damage results from static loading of a bone’s surface between the agent’s upper and lower teeth [57], [58].
2. *Tooth scores* are “essentially elongated tooth pits that are usually U-shaped in cross-section, where a consumer’s tooth cusp maintained brief contact with […] the bone surface as the tooth cusp dragged away from the pit basin” [61, p.124].
3. *Furrowing* is the chewing-driven deletion (e.g., “scooping out”) of trabeculae, usually from an LLB’s epiphysis [51], [53], [58], [62].
4. *Crushing* is produced by the collapse of localized areas of cortical tissue under the static loading of a consumer’s jaws and teeth, forming cracks and splitting in these regions [58].
5. *Crenulated edges* often occur on flat bones in the form of ragged, notched terminations imparted by a consumer in areas where its bite force overcame the structural strength and density of bone tissue [57], [58].
6. *Classic and general peeling* of bone [63] are regions of bone where cortical layers have been stripped away [64], usually by the action of a consumer’s anterior teeth; with *incipient peeling*, cortical layers are simply frayed and perhaps splayed or bent but not removed from the bone [63].

For quantitative analyses of bone damage distributions and frequencies, we divided carcasses into anatomical regions, including: the skull (cranium and mandible); axial skeleton (ribs and vertebrae); girdles (scapulae and pelves); LLBs (including humeri, radioulnae, femora and tibiae, but excluding metapodials, which are essentially meatless and thus rarely intensively modified by non-durophagus meat-eaters). Teeth and podials were excluded from most of our quantitative analyses. For overall anatomical region distribution and frequency patterns, we display our statistical analytical results on ternary graphs [65], which are based on a likelihood method that is, in turn, an improvement over traditional bootstrapping methods. With ternary graphing, the likelihood of confidence intervals does not depend on sample size [65]. An additional advantage of the likelihood method is that it can accommodate counts of zero, displayed on ternary graphs at vertices. For length and breadth (mm) of bear tooth pits, punctures and scores, we performed a Mann-Whitney test between all possible pairs of samples to determine which differed significantly, including variables such as tissue bone (cancellous and thin cortical bones), size of bear consumers and size of ungulate bones.

### Analysis of LLB modification patterns

Our more specific analytical treatment of the taphonomic alteration of LLB specimens was explicitly comparative. It is obvious that the more alike the processes and fundamental variables (e.g., environment/habitat, community structure, season, etc.) of actualistically derived taphonomic models and the paleontological/archaeological subject materials they attempt to model, the more robust is the heuristic utility of the former (e.g., [14], [66], [67]). Accordingly, we strove to control, to the greatest extent possible, as many variables as possible across our three comparative samples. First, the composition of our comparative samples is uniform, consisting only of LLB specimens. Second, all samples formed under wild, free-ranging situations, through the taphonomic actions of large, gregarious carnivores, including spotted hyenas, lions and wolves (samples formed by brown and striped hyenas are also included in our analyses, but in more minor ways). Third, we were able to largely control for carcass taxon and size by utilizing observations of samples that are composed in large parts by the bones of equids modified by our comparative carnivore subjects. Table 2 summarizes key aspects of the comparative samples we utilized in this study [4], [10], [11], [15], [67]–[72].

**Table 2:** Major features of the taphonomic samples utilized in this study.

Taphonomic agent	Study area	# carcasses	Carcass type	Carcass size class	LLB data source
Lions	Tarangire 2008	8	Equids	3	[67]
	Tarangire 2009	5	Equids	3	[67]
	Tarangire 2010	5	Equids	3	[67]
	Tarangire	42	Equids, suids, bovids, giraffids	2,3,4,5	[15]
	Tarangire	31	Equids, suids, bovids	2,3	[15]
	Tarangire	11	Bovids, giraffids	4,5	[15]
	Maasai Mara	8	Equids	3	[11]
Wolves	Monte Campelo 1	4	Equids	3	[72]
	Monte Campelo 2	5	Equids	3	[72]
	Monte Campelo 3	8	Equids	3	[72]
	Flechas	17	Equids	3	[69]
	Villadeciervos	11	Equids	3	[69]
Spotted hyenas	Maasai Mara den	8	Equids	3	[10]
	Kisima Ngeda Den 2	47	Equids, bovids	1,2,3	[70]
	Amboseli	63	Equids, suids, bovids, carnivores	1,2,3,4	[4]
Striped hyenas	Jordan	35	Bovids	2,3,4	[71]
Brown hyenas	Southern Africa	8	Bovids	2,3,4	[71]
Brown bears	Pallars Sobirà, Vall d’Aran	17	Equids, bovids	1,2,3,4	This study

Carcass size categories are modified from Bunn [68] system for grouping osteological remains according to estimated live body weights of adult animals: size class 1 = <20 kg; size class 2≈20–120 kg; size class 3≈120–300 kg; size class 4≈300–1000 kg; size class 5>1000 kg. Abbreviations: LLB = long limb bone.

For the comparative, interspecific analyses of the LLB subsamples, we focused on the following variables:

1. Operating from the hypothetical assumption that, all things being equal, durophagus carnivores are likely to produce much greater frequencies of furrowing on LLB epiphyses, we scored the simple presence or absence of furrowing on the ends of LLBs. More specifically, we conducted multivariate statistical analyses of the occurrence of furrowing on distal humeri, proximal ulnae, proximal and distal femora, and proximal tibiae.
2. We quantified tooth mark (including pits, punctures and scores) frequencies per bone specimen. A range of measures for pits and punctures has been established to allow comparison with those provided by other researchers [55], [56], [73] and on other carnivores (e.g., [36], [72]–[77]).
3. All things being equal, durophagus carnivores are predicted to destroy bone epiphyses completely in greater frequency than do non-durophagus carnivores (e.g., [78]). Therefore, for each sample, we quantified LLB completeness thusly: complete; cylinder (a LLB specimen lacking its epiphyses, represented by a portion of diaphysis that for, at least some of its length, maintains its complete, original circumference); LLB diaphysis fragment (a shaft fragment that maintains <100% of its complete, original circumference).
4. All things being equal, durophagus carnivores are predicted to impart a higher proportion of tooth pits to tooth scores on dense cortical bone than are non-durophagus, who primarily focus only on meat-stripping rather than on meat-stripping *and* bone-cracking, as do durophagus carnivores (e.g., [6]). Thus, we calculated pit: score ratios for all comparative samples.

### Statistical analysis of LLB modification patterns

In order to document relationships among analytical components of the comparative LLB samples, we conducted principle component analyses (PCA), which produce factors that result from the reduction of dimensionality caused by multiple variables. With exploratory PCA, the analyst aims to improve prediction and variance accountability by detecting those variables that do not contribute significantly to sample variance; with confirmatory PCA, the analyst uses selected variables to maximize sample variability and sample component relationships. The use of continuous numerical variables may result in bias of PCA solutions due to the heterogeneity of these values and overemphasis of the weight of variables displaying high numerical values. For this reason, variables are usually centered and scaled prior to their statistical analysis. However, in the present analysis, all variables involved the use of percentage values; they have similar scale, so there was no need to center and scale variables.

As opposed to dimension reduction by orthogonal projection as performed in PCA, in multidimensional scaling (MDS) points are chosen so that stress (the sum of the squared differences between the inter-sample disparities and the inter-point distances) is minimized [79]. The MDS option we selected for our analyses is the identity transformation, which consists of taking the inter-sample disparities as the inter-sample dissimilarities themselves. This metric MDS approach uses a Pythagorean metric analysis of inter-point distances, which includes an iterative majorization algorithm to find the MDS solution [79]. This algorithm was considered to have converged as soon as the relative decrease in stress was less than 10^−6^. The algorithm was also stopped once greater than 5,000 iterations were performed. In MDS, points are related in a low-dimensional Euclidean space [79], with data spatially projected by regression methods admitting non-linearity. The use of MDS in the present study therefore complements the PCA test.

We also employed a canonical variate analysis (CVA). CVA focuses on data grouped into K classes by transforming original variables into canonical variables defined by square distances between the means of the groups obtained by Mahalanobiśs D^2^. This is scale invariant. CVA produces a higher degree of separation between the group means than does either PCA or MDS [80]. The biplot axes for CVA were determined by the group means.

In a separate database, we entered LLB completeness data (see above, point 3) and tooth score/tooth pit ratio (see above, point 4) data. Both data sets were analyzed together using a Redundancy Analysis (RDA), which combines a multiple regression with a PCA. Data were previously normalized using a Hellinger transformation method [81]. Missing data were converted using group average values.

Because results of the multivariate statistical analyses summarized above differentiated our comparative carnivore groups (see below Results), our next step was to create thresholds from which frequencies of different modification types could be used as lines to demarcate the different types of carnivoran taphonomic agents. To this end, we submitted data for all taxa and all variables to a tree-based analysis.

Regression and classification trees usually suffer from high variance, but a procedure with a repeated sequence of data sets derived from the same original sample will decrease that variance. This is the general purpose of bootstrap aggregation, more commonly known as bagging, which splits the original training data set (TDS) into multiple data sets derived from bootstrapping the original TDS. Similarly, the powerful procedure of random forests (RF) performs a bootstrapping approach, but samples only a subset of variables for each tree. Thus, RF produces a final solution that includes a selection of variables that are important for correct classification of the analytical set. RF produces hundreds of trees that are repeatedly fitted to bootstrapped sets of data. The results are contrasted against a validation test, from the observations (about one third) not used for the training data set (these observations are referred to as out-of-bag (OOB) observations). RF produce estimates on how many iterations are needed to minimize the OOB error. The importance of each response variable is determined by mean decreased error (MDE) for regression trees (RT), whereas the Gini index is more useful for classification trees (CT). The following step is the creation of a classification tree using the most useful variables selected by the RF test.

We conducted an RF test by selecting three-variable sets in each aggregated bootstrap iteration [10], [15], [69], [70]. A total of 500 trees was generated. The test was applied to all variables in Table 3. Subsequently, it was applied only to the variables dealing with LLB portion modification. Variable selection for CT analysis was carried out when variables showed a mean decreased Gini index of >0.5.

**Table 3:** Frequencies of long limb bone (LLB) portions (P = proximal; D = distal) with tooth mark damage, tooth pit/tooth score ratios and frequencies of LLB cylinders and complete LLBs in comparative faunal samples (data sources listed in Table 2; data for brown bears are from this study).

Taphonomicagent	Sample	P. Humerus(%)	D. Humerus(%)	P. Radius(%)	D. Radius(%)	P. Femur(%)	D. Femur(%)	P. Tibia(%)	D. Tibia(%)	Pit:Score	Cylinders(%)	Complete(%)
Lions	Tarangire 2008	81	40	–	–	64	64	20	–	0.25	–	100
	Tarangire 2009	87.5	25	–	–	16	50	–	–	0.25	–	100
	Tarangire 2010	70	10	–	–	66	78	25	–	0.25	–	100
	Tarangire, all taxa	80.9	26.1	–	10.2	78.5	6.6	39.7	9.5	0.4	–	91.6
	Tarangire, small-medium taxa	79.4	25	–	12	82.8	75	49	12.7	0.42	–	89
	Tarangire, large taxa	87.5	31.2	–	5	65	40	11.1	-	0.38	–	94
	Masaai Mara	75	18	–	–	75	19	18	–	0.47	–	88
Wolves	Monte Campelo 1	20	–	16	16	14	43	–	–	1.5	2.7	92
	Monte Campelo 2	16	–	–	–	30	10	20	–	1.9	–	98
	Monte Campelo 3	100	43	57	43	74	82	57	33	1.2	39.5	44
	Flechas*	45.5	–	22	11	82	73	60	–	1.6	3.3	86.9
	Villadeciervos*	80	–	–	–	80	59	66.7	–	1.6	7.7	88.5
Spotted hyenas	Masaai Mara den	75	50	45	56	80	80	80	20	1.2	30.6	23
	Kisima Ngeda Den 2	100	75	-	67	33	63	33	–	1.3	16	29
	Amboseli	100	64.5	15.4	72	66.6	71.4	60	45.8	–	–	–
Striped hyenas	Jordan	40	60	34.7	34.7	54.6	72.7	46	37.8	–	6.0	–
Brown hyenas	Southern Africa	100	75	–	25	100	50	20	–	–	10.0	–
Brown bears	*Bos* and *Equus*	–	–	–	–	–	66.6	50	–	0.5	–	100
	*Cervus*	57.1	–	–	–	28.6	14.3	50	–	3.3	–	100
	Small ungulates	40	–	–	–	20	20	–	–	1.8	–	98

*Tooth mark data obtained from conspicuous damage on ends alone.

The variables with higher MDE values were then selected to obtain single regression trees. CT tests were carried out by selecting a complexity parameter (cp) of 0.005.

The frequency data are shown in Table 3. Analyses were performed in R. The PCA, CVA, and MDS tests are graphically displayed as biplots using the R library “BiplotGUI.” RDA was performed with the aid of the “ade4”, “vegan”, “packfor”, “MASS”, “ellipse” and “FactoMineR” libraries and variables and data were plotted in a triplot. RF analysis was carried out with the R library “rattle”, which uses the “randomForest” library for RF tests and the “rpart” library for RT tests.

## Results and Discussion

### Patterns of carcass consumption by brown bears

Table 1 summarizes many relevant details of the 17 ungulate carcass occurrences modified by bears. In general, feeding bears rarely moved carcasses any appreciable distances, if at all. Most carcasses maintained near natural anatomical position and connectivity of skeletal elements upon recovery by our team. In the rare instances of distarticulation and disassociation of parts (between 50 to 250 m^2^ from a main carcass cluster), we note that much of this separation might be explained the actions of non-bear, secondary consumers, such as foxes (*Vulpes vulpes*), martens (*Martes martes*) and vultures (*Gyps fulvus*, *Gypaetus barbatus*)–all of which are active in the region (Observations [OB] 4–7). Minor water flows could have also carried remains away from carcass concentrations (OB 17). The very incidental movement of carcasses by bears most probably relate to the minor agitations caused by infrared lights on the photo- and videotraps and human scents in the vicinities of carcasses. But, in agreement with the observations of Sala and Arsuaga [56] in Cantabria, the Pyrenean brown bears did not transport carcasses or carcass parts to dens or other shelter sites. Although Cantabria is home to wolves, like us, Sala and Arsuaga [56] did not observe large, non-ursid carnivores influencing bear feeding behavior. In more intensive competitive situations bears might be more likely to transport and secure carcasses in shelter refuges. Thus, as it now stands, neotaphonomic observations of extant brown bears hold little relevance for modelling potential carcass transport by extinct ursids, which are sometimes implicated in the substantial accumulation of European Pleistocene cave faunas.

**Figure 2:**
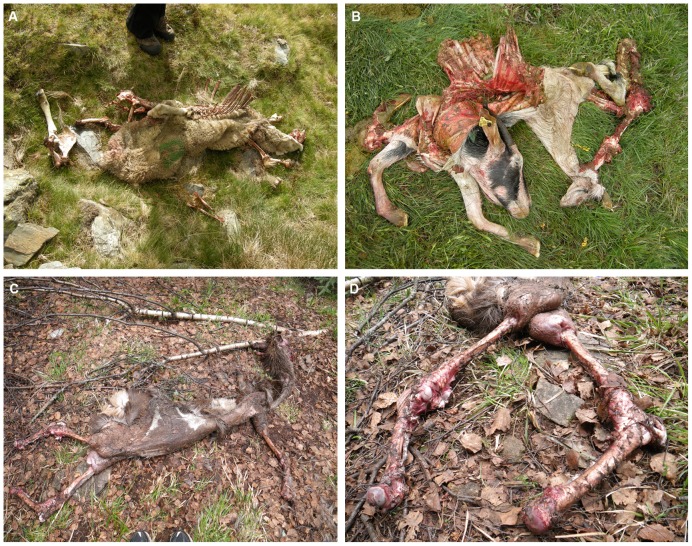
Examples of instances in which bears removed the skin of carcasses before feeding. A) Observation (OB) 14; B) Observation (OB) 12; C) Observation (OB) 4; D) detail of hind limb from Observation 4. Note that hind limbs were skinned and attached to the fur only by autopodials.

**Figure 3:**
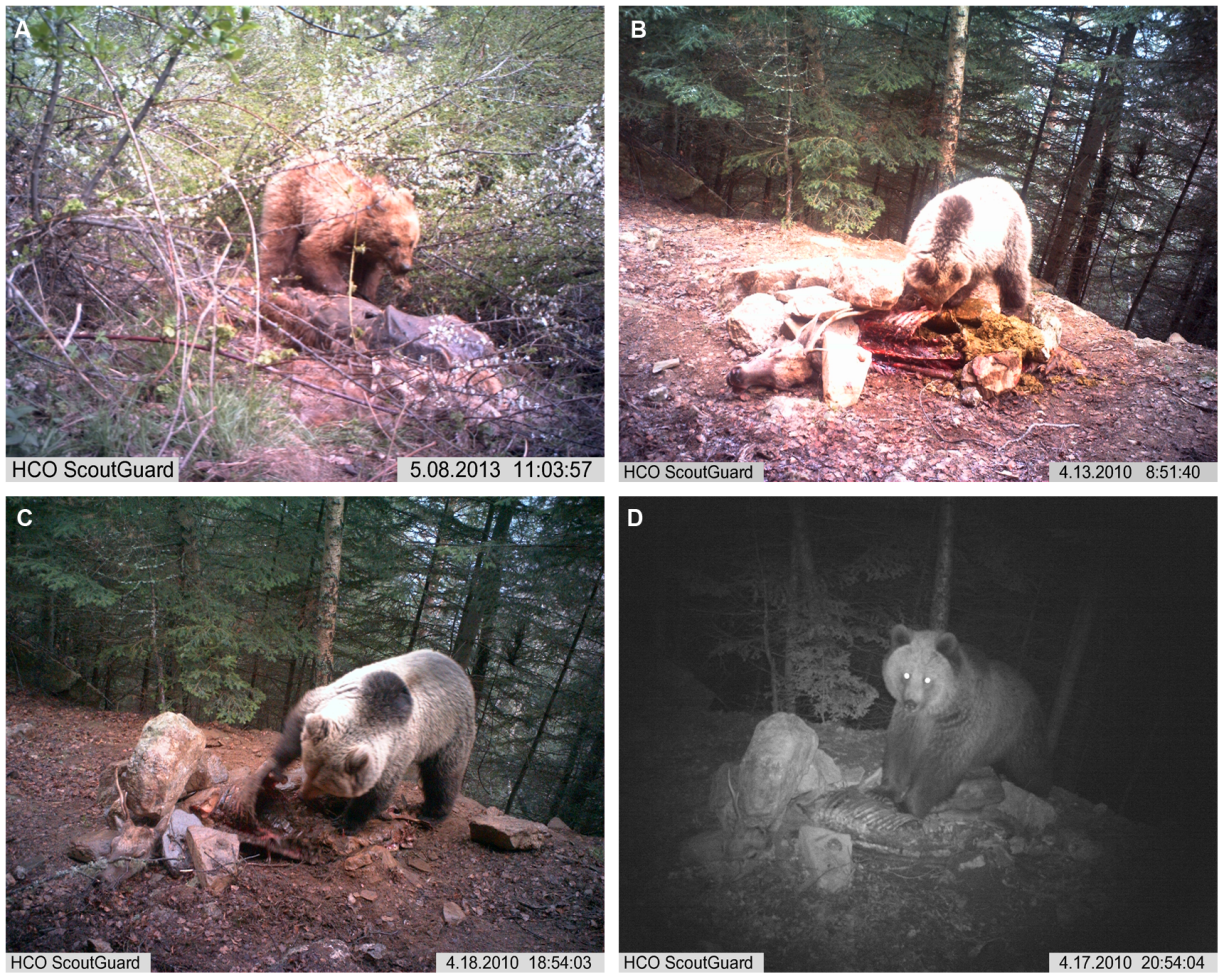
Some actions related to bear breakage of ungulate axial bones. A) Small bear pressing down on the ribcage of a red deer; B) large bear breaking ribs by sudden jerking of head, with his teeth clamped onto the carcass; C) large bear expanding the ribcage of a red deer; D) large bear pressing down on the ribcage of a red deer.

In nine instances (OB 4, 8–14, 17), bears skinned carcasses before consumption (see also, [50], [82] (Figure 2). All cases of skinning involved small sized carcasses; no large carcasses were completely skinned. An example of partial skinning by a bear is shown in Video S1, in which a young bear consumes the viscera of an adult male deer without removing the carcass’s whole skin. Skinning seems to have facilitated separation of a carcass’s trunk and limbs, after which the trunk’s viscera was removed and consumed. We do not associate any subsequent superficial modifications on recovered bones to skinning by bears. Across carcass sizes, removal and consumption of viscera usually commenced at the abdomen of the carcass, with bears employing their forepaws to wrench back ribs dorsally, away from their sternal articulations. In only one case (OB 7), involving the consumption of a large (>500 kg) cow carcass, did a bear initiate removal of viscera differently, by moving from the carcass’s throat in a caudal direction toward the lower abdomen and *viceversa*. To deal with this large carcass, the bear consumer took a multistep tact to opening the cow’s ribcage, as is viewable in Video S2. The bear initiated the process by compressing the cow’s ribcage under its forepaws and body weight, fracturing ribs. Next, the bear pried apart the ribcage with its forepaws, and then bit the sternal ends of ribs, separating them with brisk jerking of its head and the assistance of its forepaws (some examples of these actions are shown in Figure 3). In parallel, some portions of musculature covering vertebrae and pelves, and to a lesser extent upper LLBs, are defleshed and consumed (OB 1, 2, 6, 8, 11, 13–16).

Following the consumption of viscera and meat, bears focused on chewing-directed extraction of intrabone nutrients from especially the proximal epiphyses of humeri and tibiae, both epiphyses of femora, from the innominates and from the bodies of vertebrae. Sala and Arsuaga [56] observed similar behaviors among the Cantabrian bears they studied. However, unlike the observations of Sala and Arsuaga [56], as well as those of Haynes [53] and Pinto and Andrews [83], the Pyrenean bears did not fracture open LLB diaphyses for access to yellow marrow. Unique among bear studies, we *did* observe an instance of a bear breaking open a deer spine along the lumbar section in order to gain access to edible spinal cord tissues (Video S3).

### Overview of the taphonomy of the brown bear-derived sample

**Figure 4:**
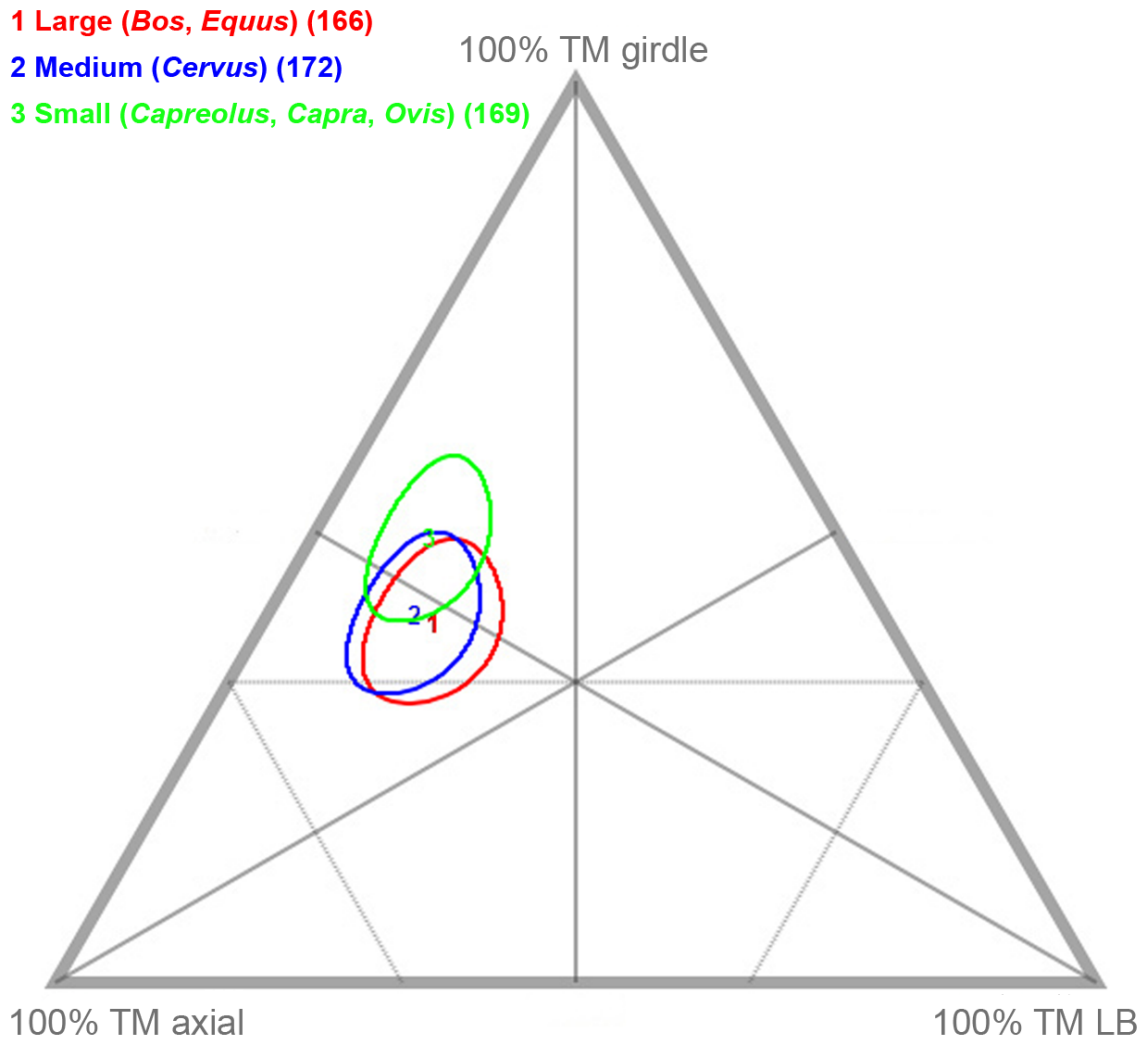
Ternary-plot showing bone damage classified by ungulate anatomical units.

The aggregated osteological sample from all 17 ungulate carcasses consumed by bears shows a variety of consumption-related damage, including tooth punctures, pits and scores, crushing, the furrowing of LLB epiphyses, crenulation, fracturing and peeling (Table 4). Of the total recovered number of identified specimens (NISP) of 1,173, 508 (43.3%) bear at least one occurrence of taphonomic modification, with most of the damaged specimens deriving from bones of the axial (NISP = 459) and girdle (n = 22) regions (Tables 5, 6; Figure 4). Only five skull specimens and 21 LLB specimens (as well a single patella) preserve bear-induced modifications. Ungulate carcass size seems to have had only minimal influence on divergences from these general patterns of assemblage-level modification, with slightly more intensive damage observed on scapulae and pelves derived from smaller carcasses (Table 4).

**Table 4:** Brown bear induced-damage by ungulate body size (see text for definitions) and skeletal element.

Skeletal element	Size categories	Recovered bone specimens	Damaged bone specimens	Pits/Scores		Furrowing		Crenulated edge		Transverse fractures		Crushing		Longitudinal cracks		Peeling (classic)		Peeling (incipient)	
		n	%	n	%	n	%	n	%	n	%	n	%	n	%	n	%	n	%
Cranium	Small	8	37.50	3	37.50	1	12.50	–	–	–	–	1	12.50	–	–	–	–	–	–
Hemimandible	Large	4	50.00	–	–	2	50.0	–	–	–	–	–	–	–	–	–	–	–	–
Cervical vertebra	Large	13	38.46	1	7.69	1	7.69	–	–	2	15.38	3	23.08	–	–	–	–	–	–
	Medium	21	85.71	6	28.57	4	19.05	2	9.52	8	38.10	11	52.38	–	–	–	–	–	–
	Small	48	45.83	7	14.58	5	10.42	4	8.33	6	12.50	11	22.92	–	–	1	2.08	–	–
Thoracic vertebra	Large	22	86.36	9	40.91	3	13.64	2	9.09	8	36.36	5	22.73	1	4.55	1	4.55	–	–
	Medium	46	67.39	7	15.22	–	–	5	10.87	21	45.65	10	21.74	–	–	–	–	–	–
	Small	105	60.00	10	9.52	2	1.90	6	5.71	38	36.19	5	4.76	1	0.95	11	10.48	1	0.95
Lumbar vertebra	Large	6	100.0	–	–	–	–	–	–	6	100.0	1	16.67	–	–	3	50.0	–	–
	Medium	19	94.74	4	21.05	–	–	1	5.26	13	68.42	2	10.53	1	5.26	3	15.79	–	–
	Small	61	91.80	10	16.39	–	–	8	13.11	22	36.07	7	11.48	–	–	25	40.98	7	11.48
Caudal vertebra	Small	3	33.33	1	33.33	–	–	–	–	–	–	1	33.33	–	–	–	–	–	–
Sternum	Medium	3	33.33	1	33.33	1	33.33	–	–	–	–	–	–	–	–	–	–	–	–
Sacrum	Large	1	100.0	–	–	1	100.0	–	–	–	–	–	–	–	–	–	–	–	–
	Medium	3	100.0	–	–	–	–	–	–	–	–	2	66.67	–	–	–	–	–	–
	Small	8	62.50	5	62.50	2	25.00	2	25.00	–	–	2	25.00	–	–	–	–	–	–
Rib	Large	23	78.26	13	56.52	–	–	–	–	9	39.13	5	21.74	1	4.35	1	4.35	–	–
	Medium	77	76.62	22	28.57	–	–	3	3.90	26	33.77	12	15.58	–	–	7	9.09	18	23.38
	Small	187	71.12	47	25.13	–	–	8	4.28	55	29.41	26	13.90	7	3.74	22	11.76	32	17.11
Scapula	Large	4	50.00	2	50.0	–	–	–	–	–	–	–	–	–	–	–	–	–	–
	Medium	4	50.00	–	–	–	–	1	25.00	–	–	2	50.0	–	–	–	–	–	–
	Small	8	50.00	1	12.50	–	–	3	37.50	–	–	1	12.50	–	–	2	25.0	–	–
Humerus	Medium	7	57.14	3	42.86	4	57.14	1	14.29	–	–	–	–	–	–	–	–	–	–
	Small	5	40.00	1	20.0	2	40.0	–	–	–	–	–	–	–	–	–	–	–	–
Ulna	Large	1	100.0	–	–	1	100.0	–	–	1	100.0	–	–	–	–	–	–	–	–
	Medium	7	14.29	–	–	–	–	–	–	–	–	1	14.29	–	–	–	–	–	–
	Small	5	60.00	2	40.0	–	–	–	–	1	20.0	–	–	–	–	–	–	–	–
Innominate	Large	2	100.0	2	100.0	1	50.0	–	–	1	50.0	–	–	–	–	–	–	–	–
	Medium	6	83.33	4	66.67	2	33.33	–	–	–	–	3	50.0	–	–	–	–	–	–
	Small	10	70.00	5	50.0	1	10.0	3	30.0	–	–	4	40.0	–	–	–	–	–	–
Femur	Large	3	66.67	1	33.33	2	66.67	1	33.33	–	–	–	–	–	–	–	–	–	–
	Medium	4	75.00	2	50.0	3	75.00	–	–	–	–	–	–	–	–	–	–	–	–
	Small	5	20.00	–	–	1	20.0	–	–	–	–	–	–	–	–	–	–	–	–
Tibia	Large	4	50.00	–	–	2	50.0	–	–	–	–	–	–	–	–	–	–	–	–
	Medium	4	50.00	–	–	2	50.0	–	–	–	–	–	–	–	–	–	–	–	–
Patella/Sesamoid	Medium	30	3.33	–	–	1	3.33	–	–	–	–	–	–	–	–	–	–	–	–
Total*			43.31	169	14.41	44	3.75	50	4.26	217	18.50	115	9.80	11	0.94	76	6.48	58	4.94

Note that some specimens show co-occurrence of damage and therefore, the total number (summing up all types) can be higher in some categories. *The percentages were calculated on the total number of recovered bones (*n* = 1,173–see Table 5 for more detail).

**Table 5:** Numbers of recovered ungulate skeletal elements and damaged bones in the aggregated bear-modified assemblage (by observation [OB]).

Skeletal element	# recovered (# damaged)																	
	OB1^ S^	OB2^ UNK^	OB3^ UNK^	OB4^ S^	OB5^ UNK^	OB6^ S^	OB7^ S^	OB8^ P^	OB9^ P^	OB10^ P^	OB11^ P^	OB12^ P^	OB13^ P^	OB14^ P^	OB15^ S^	OB16^ S^	OB17^ P^	Total
Cranium	1	–	–	1	1	–	1	1	1(1)	1	–	1(1)	1	1	1	–	1(1)	12(3)
Hemimandible	2(2)	–	–	2	2	2	2	–	–	2	–	–	2	2	2	–	–	18(2)
Atlas	1(1)	–	–	1(1)	1(1)	–	–	1	–	1(1)	–	1(1)	1	1	1	–	1	10(5)
Axis	1(1)	–	–	1(1)	1(1)	1(1)	1	1	1	1(1)	–	1(1)	1	1	1(1)	1(1)	1	14(8)
Cervical	5(3)	–	–	–	1(1)	5(5)	5	5	1(1)	5(5)	2(2)	4(4)	5	5(1)	5(4)	5(4)	5(2)	58(32)
Thoracic	12(10)	11(8)	2(2)	9(5)	13(13)	11(11)	10(9)	13	1(1)	13(1)	13(11)	–	13(1)	13(13)	13(9)	13(6)	13(13)	173(113)
Lumbar	6(6)	6(6)	6(6)	2(2)	6(6)	6(6)	–	7(3)	1(1)	7(7)	7(7)	3(3)	6(5)	6(6)	5(5)	6(5)	6(6)	86(80)
Sacrum	1(1)	1	1(1)	–	1	1(1)	–	–	–	1(1)	1(1)	–	1	1(1)	1(1)	1(1)	1(1)	12(9)
Caudal	–	2	–	–	–	–	–	–	–	1(1)	–	–	–	–	–	–	–	3(1)
Sternum	–	–	–	3(1)	–	–	–	–	–	–	–	–	–	–	–	–	–	3(1)
Rib	7(7)	22(22)	1(1)	16(14)	25(18)	14(14)	16(11)	24(2)	2(2)	21(19)	23(22)	6(6)	26(6)	20(19)	26(14)	21(17)	17(16)	287(210)
Scapula	2(2)	–	–	2	2(2)	1(1)	2	2	–	–	–	2(2)	2	–	1(1)	–	–	16(8)
Humerus	1	–	–	2(2)	2(1)	2(2)	2	–	–	1(1)	–	–	2	–	2	1	–	15(6)
Radius	1	–	–	2	2	2	–	–	–	1	–	–	2	–	2	1	–	13
Ulna	1(1)	–	–	2(1)	2(2)	2	–	–	–	1(1)	–	–	2	–	2	1	–	13(5)
Carpals	14	–	–	17	14	14	10	–	–	6	–	–	12	–	14	5	–	106
Metacarpal	2	–	–	2	2	2	2	–	–	1	–	–	2	–	2	1	–	16
Innominate	2(2)	2(2)	–	–	2(2)	2(2)	–	2	–	–	–	–	2(1)	2(2)	2(1)	2(2)	–	18(14)
Femur	1(1)	–	–	2(2)	2	–	2(1)	–	–	–	–	1(1)	2	–	2(1)	–	–	12(6)
Tibia	2(2)	–	–	2(2)	1	–	2	–	–	–	–	–	2	–	2	–	–	11(4)
Patella/Sesamoids	8	–	–	9(1)	12	–	6	–	–	4	–	–	10	–	17	4	–	70(1)
Tarsals	12	–	–	7	4	1	3	–	–	–	–	–	10	–	10	1	–	48
Metatarsal	2	–	–	2	1	1	–	–	–	–	–	–	2	–	2	–	–	10
Lateral metapodial	–	–	–	–	4	–	–	–	–	–	–	–	–	–	–	–	–	4
Phalanges	12	–	–	24	18	12	5	–	–	6	–	–	20	–	24	6	–	127
Accessory phalanges	–	–	–	–	14	–	–	–	–	–	–	–	4	–	–	–	–	18
Total	96(39)	44(38)	10(10)	108(32)	133(47)	79(43)	69(21)	56(5)	7(6)	73(38)	46(43)	19(19)	130(13)	52(42)	137(37)	69(36)	45(39)	1,173(508)

Superscripts refer to the type of access to carcasses by bears: S =  Scavenging (OBs 1, 6 and 7 correspond to ungulate natural deaths and; OBs 4, 15 and 16 to Roadkills); P =  Predation (OBs 8–14 and 17); UNK =  Unknown (OBs 2, 3 and 5) (see Table 1 for more details).

**Table 6:** Numbers of recovered ungulate skeletal elements and damaged bones in the aggregated bear-modified assemblage (by observation [OB] and anatomical segments [defined in the text]).

Size categories	Carcass	Observation	Recovered bones							Damaged bones (%)						
			Skull	Axial	Girdle	Limbs	Basipod	Acropod	Pat/Sesam	Skull	Axial	Girdle	Limbs	Basipod	Acropod	Pat/Sesam
Large size	Horse	OB 1^S^	3	33	4	10	26	12	8	2 (66.67)	29 (87.88)	4 (100.0)	4 (40.00)	–	–	–
	Cattle	OB 7^S^	–	42	2	–	–	–	–	–	36 (85.71)	2 (100.0)	–	–	–	–
Medium size	Red deer	OB 4^S^	–	10	–	–	–	–	–	–	10 (100.0)	–	–	–	–	–
		OB 6^S^	3	32	2	14	24	24	9	–	24 (75.00)	–	7 (50.00)	–	–	1 (11.11)
		OB 15^S^	3	48	4	12	18	32	16	–	40 (83.33)	4 (100.0)	3 (25.00)	–	–	–
		OB 16^S^	2	38	3	9	15	12	–	–	38 (100.0)	3 (100.0)	2 (22.22)	–	–	–
Small size	Roe deer	OB 2^UNK^	3	32	2	8	13	5	6	–	20 (62.50)	–	1 (12.50)	–	–	–
		OB 3^UNK^	1	51	4	–	–	–	–	–	5 (9.80)	–	–	–	–	–
		OB 5^UNK^	1	6	–	–	–	–	–	1(100.0)	5 (83.33)	–	–	–	–	–
	Sheep	OB 8^P^	3	50	–	4	6	6	4	–	36 (72.00)	–	2 (50.00)	–	–	–
		OB 9^P^	–	46	–	–	–	–	–	–	43 (93.48)	–	–	–	–	–
		OB 10^P^	1	15	2	1	–	–	–	1(100.0)	15 (100.0)	2 (100.0)	1 (100.0)	–	–	–
		OB 11^P^	3	53	4	14	22	24	10	–	12 (22.64)	1 (25.00)	–	–	–	–
		OB 13^P^	3	52	3	14	24	24	17	–	34 (65.38)	2 (66.67)	1 (7.14)	–	–	–
		OB 14^P^	–	47	2	4	6	6	4	–	34 (72.34)	2 (100.0)	–	–	–	–
		OB17^P^	1	44	–	–	–	–	–	1(100.0)	38 (86.36)	–	–	–	–	–
	Goat	OB 12^P^	3	47	2	–	–	–	–	–	40 (85.11)	2 (100.0)	–	–	–	–
Total			30	646	34	90	154	145	74	5(16.67)	459 (71.05)	22 (64.71)	21 (23.33)	–	–	1 (1.35)

Abbreviations: Basipod = basipodials; Acrop = acropodials; Pat/Sesam = patella/sesamoids. Superscripts refer to the type of access to carcasses by bears: S =  Scavenging (OBs 1, 6 and 7 correspond to ungulate natural deaths and; OBs 4, 15 and 16 to Roadkills); P =  Predation (OBs 8–14 and 17); UNK =  Unknown (OBs 2, 3 and 5) (see Table 1 for more details).

**Figure 5:**
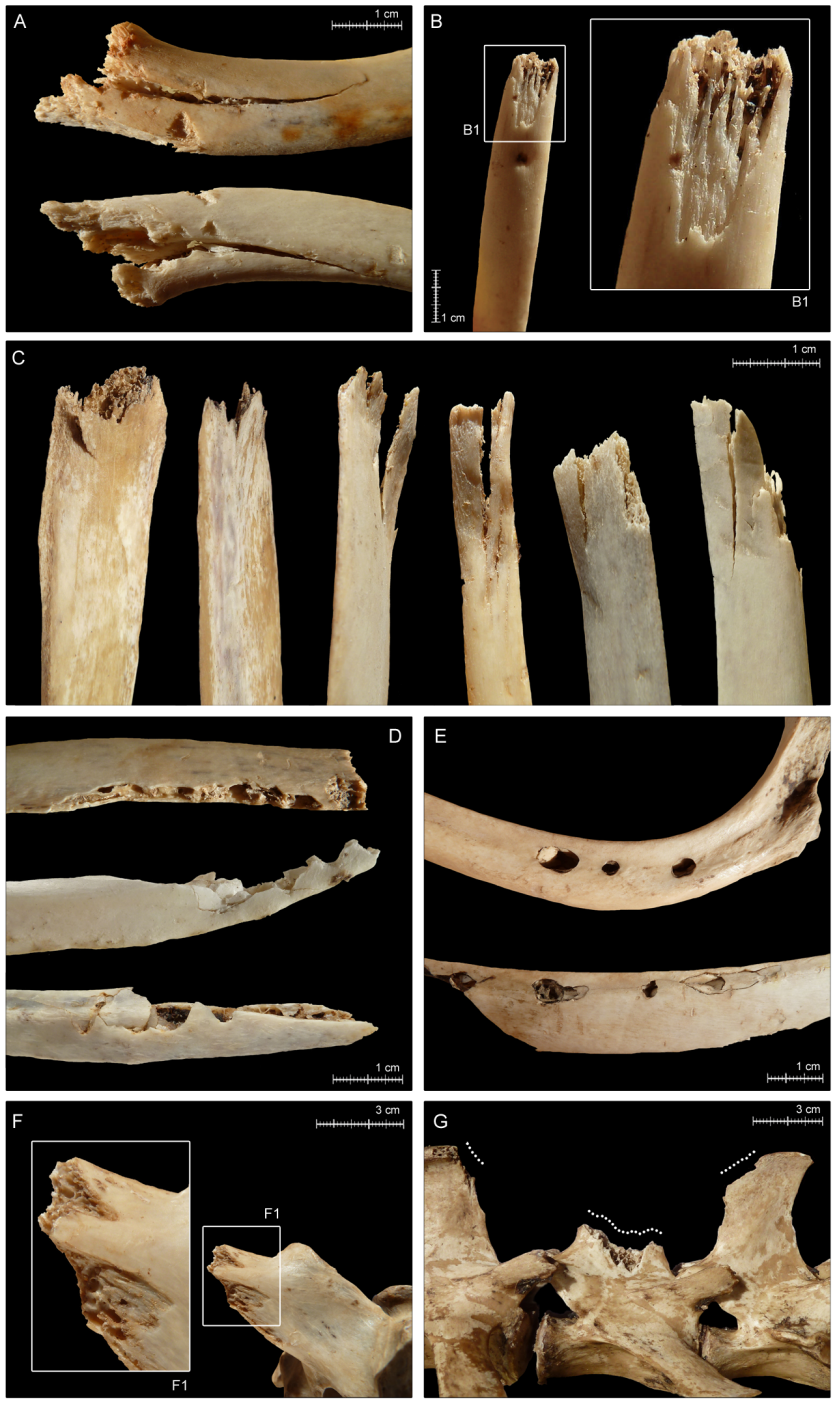
Brown bear-induced damage on axial bone specimens. A) Tooth-marked goat rib showing longitudinal cracks (OB 12); B) classic peeling on the sternal end of a roe deer rib (OB 2); C) bent ends and fraying (incipient peeling) on roe deer and red deer ribs (OB 2, 4, 5); D) crenulated edges on red deer (OB 6, 15) and sheep (OB 10) ribs; E) punctures on the vertebral end and mid-shaft of red deer ribs (OB 15); F) peeled spinous process of a sheep vertebra (OB 17); G) roe deer lumbar vertebrae showing transverse fractures (OB 5). Note the co-occurrence of classic peeling and tooth pit on the same specimen in (B).

**Figure 6:**
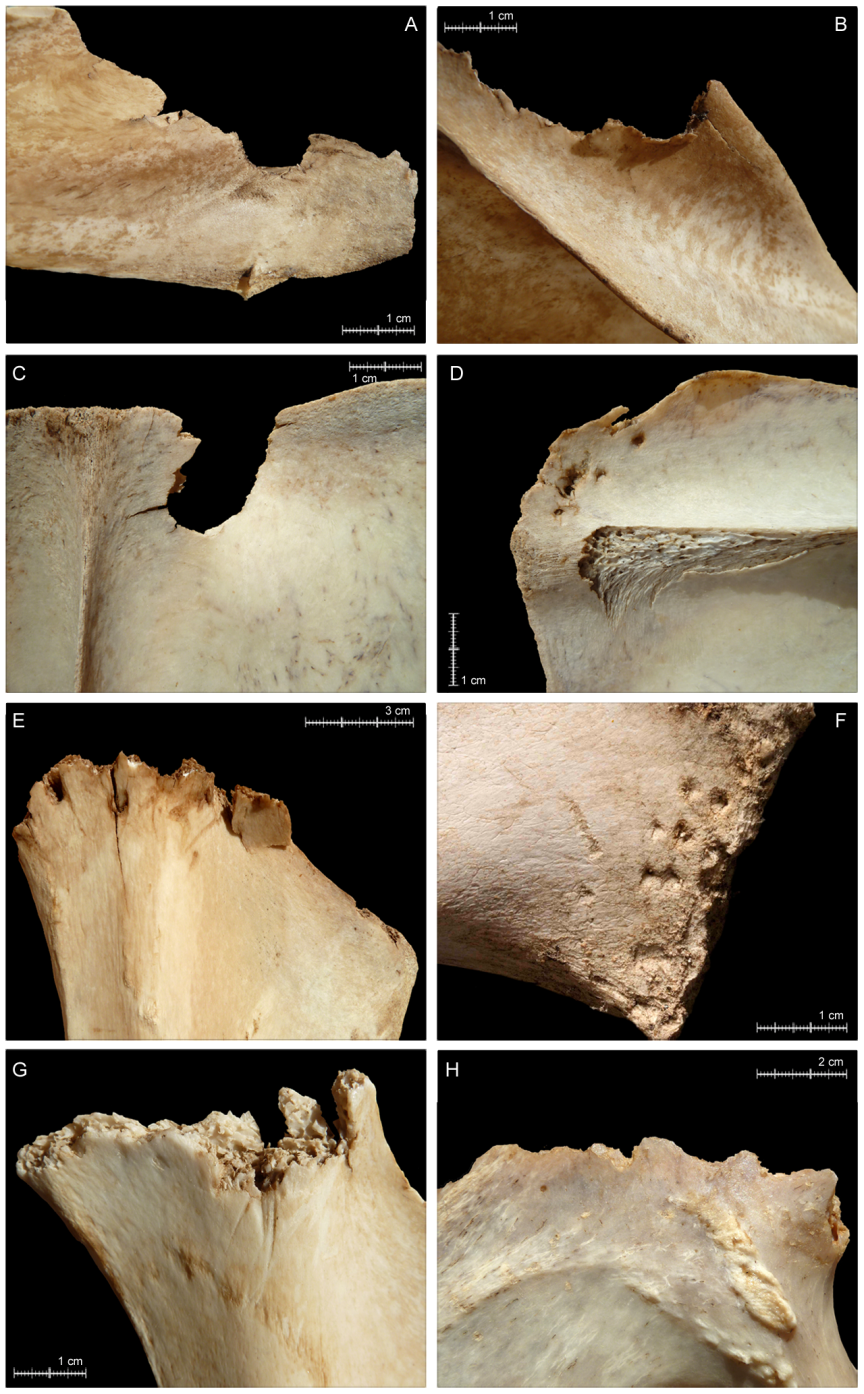
Brown bear-induced damage on girdle specimens. A–B) Crenulated edges on roe deer scapula (OB 5); C) crenulated edge on a goat scapula (OB 12); D) classic peeling and associated tooth marks on a goat scapula (OB 12); E-G) tooth marks on the innominates of a red deer (OB 16), a horse (OB 1) and a roe deer (OB 2); H) crenulated edge on a sheep ischium (OB 14).

**Figure 7:**
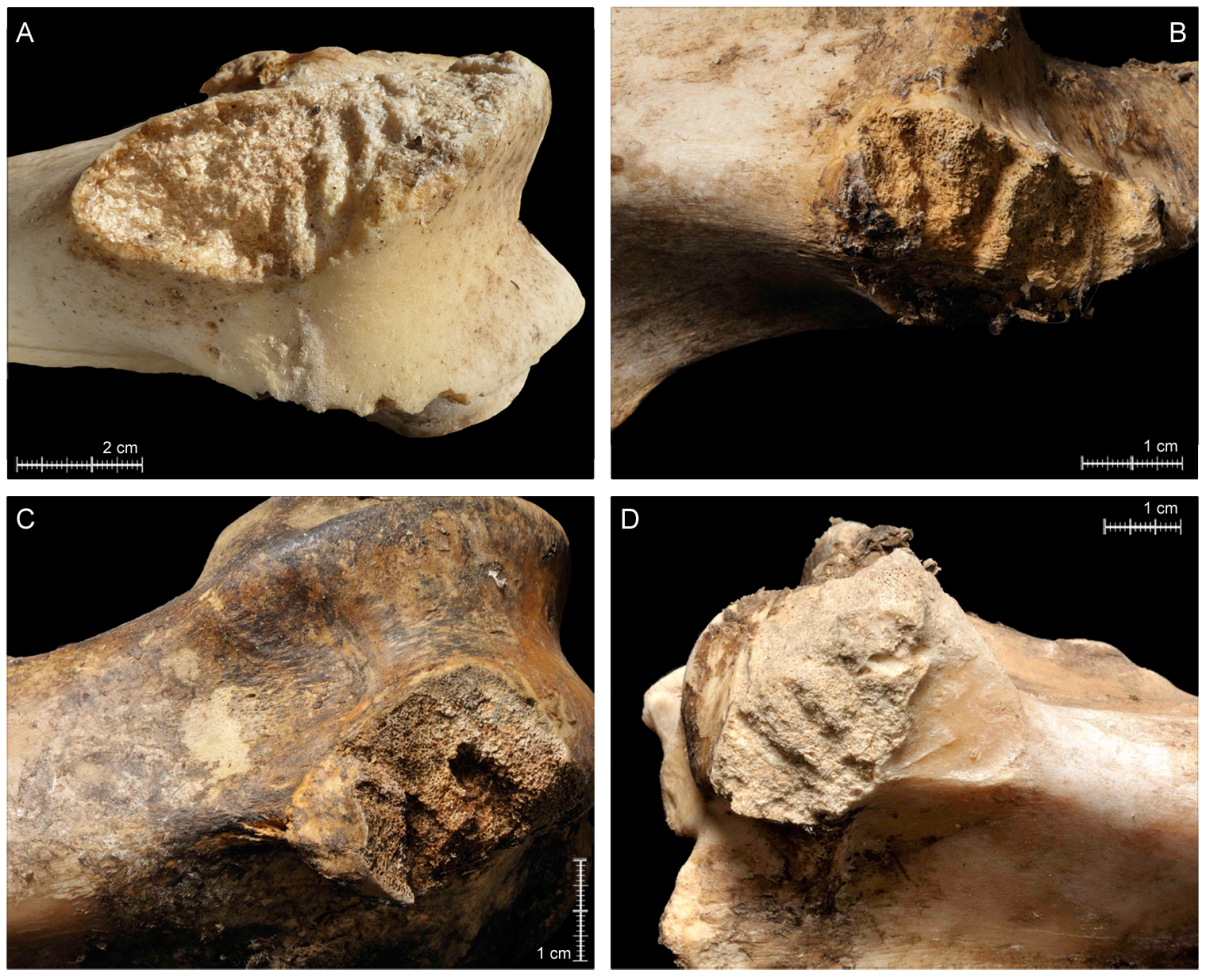
Examples of furrowing on proximal and distal epiphyses of ungulate long limb bones. A) Distal epiphysis of cow femur (OB 7); B) proximal epiphysis of horse femur (OB 1); C) distal epiphysis of horse femur (OB 1); D) proximal epiphysis of horse tibia (OB 1).

**Figure 8:**
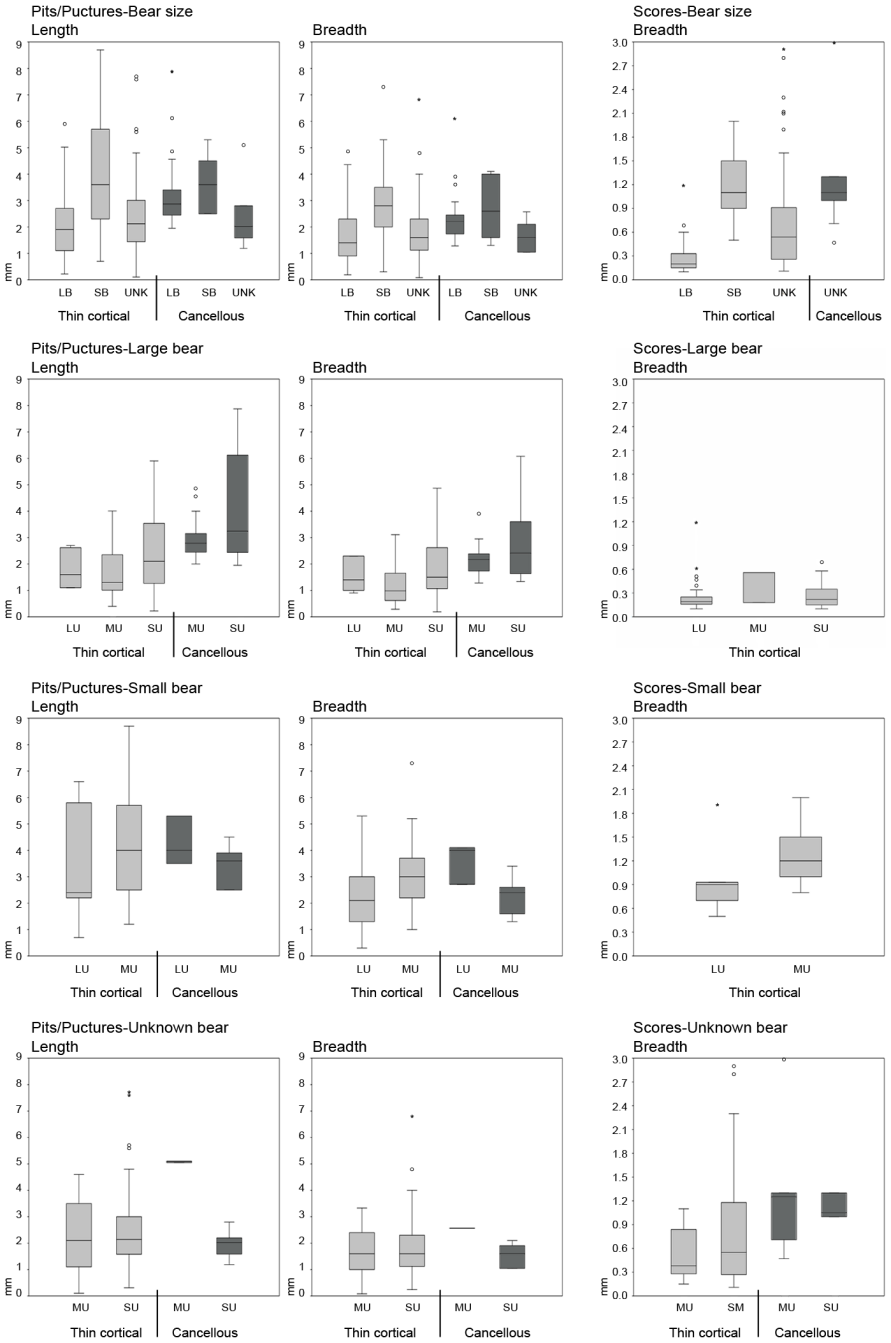
Dimensions (mm) of bear tooth pits, punctures and scores (upper and lower fence) occurring on cancellous bone and on thin cortical bone, plotted by size of bear consumers and size of ungulate bones (see Table S1 for descriptive statistics). LB =  Large bear; SB =  Small bear; Unknown bear =  UNK; LU =  Large ungulate; MU =  Medium ungulate; SU =  Small ungulate.

Here we summarize the general patterns of bone surface modification (BSM) frequencies and anatomical distributions:

1. *Transverse fractures* and *peeling damage* are two of the most common types of BSMs in the aggregated bear-modified sample (Figure 5). Both types of BSM occur predominantly on axial elements; the only exceptions being a *fractured* scapular neck and olecranon process of an ulna from a roe deer (OB 5) and a *fractured* horse ilium (OB 1).
*Transverse fractures* are the single most abundant type of BSM in the sample, occurring on 217 (18.5%) of the total NISP. Most *fractures* are observed on vertebrae (NISP = 124), including on especially lumbar vertebrae and specifically on lumbar transverse and spinous processes, and on ribs (NISP = 90) (Figure 5G). *Fractures* resulted primarily from evisceration-related activities, when ribcages were detached from sterna and pried apart by bears.
  As with *fractures*, we associate most *peeling* damage with evisceration and wrenching of ribcages by bears. One hundred thirty-four specimens (11.4% of the total aggregated NISP) are *peeled*. The most common type of *peeling* is *classic peeling* (NISP = 76), which is seen especially prominently on lumbar vertebrae (NISP = 31), with much damage on transverse processes and much more rarely on spinous processes (Figure 5F). Fewer (NISP = 12) thoracic vertebrae show *classic peeling*, with most of it on spinous processes. Only two goat scapulae show this type of modification –on portions of the coracoid process (OB 12) (Figure 6D). A NISP of 30 ribs are also *classically peeled*, with most of that damage clustered at rib sternal ends and midshafts (Figure 5B). A total NISP of 58, including 50 ribs of small and medium sized ungulates and seven lumbar vertebrae of small ungulates are *incipiently peeled* (Figure 5C).
  Our observations of *peeling* damage in the bear-modified sample are especially significant, given that this is type of taphonomic damage was heretofore primarily associated with the feeding activities of hominins (e.g., [63], [64], [84]) and chimpanzees (*Pan troglodytes*) (e.g., [85]–[87]). Anecdotal evidence suggested that other taphonomic agents, including spotted hyenas (Gary Haynes, personal communication, 2013, in [63]), also occasionally peel bones, but to our knowledge it is still relatively uncommon in non-primate-produced faunas (e.g., [63]). Thus, the high frequency of *peeling* produced by bears was unexpected, especially considering its apparent lack of occurrence in previously studied bear-created faunas, including that from Cantabria, described in great detail by Sala and Arsuaga [56]. We note that *peeling* is distributed primarily on bone specimens from small ungulates (NISP = 101) in our sample, and that, in contrast, Sala and Arsuaga [56]’s sample comprises large carcass remains; this distinction might be reason for the disparity in inter-assemblage observations of *peeling* damage.
2. *Crushing* damage is distributed broadly across skeletal parts, including on 25 cervical, 20 thoracic and 10 lumbar vertebrae of large, small and medium ungulates, on the caudal vertebra of a sheep (OB 10), and on three scapulae, seven innominates and four sacra of small and medium carcasses. C*rushing* is also observed on the nasal bones of a goat (OB 9), on the olecranon process of a red deer ulna (OB 4). Among the damaged vertebrae, *crushing* is identified mainly on the spinous (NISP = 31; 55.3%) and transverse (NISP = 16; 28.6%) processes. Occasionally, this modification is detected on mammillary apophyses (NISP = 5; 8.9%) and on the vertebral body (NISP = 4; 7.1%). Several ribs (NISP = 43) are *crushed*, including on both ends, and on necks and midshafts. Given its wide anatomical distribution, *crushing* is associated with both ribcage opening activities and general feeding/chewing.
3. *Longitudinal cracking* of specimens is relatively rare in the aggregated bear-derived sample (NISP = 11), but clusters in the axial region and is thus probably related to prying and wrenching of ungulate thoraxes when bears sought access to viscera (Figure 5A). Seven rib specimens from small ungulates and one from a large ungulate are *cracked*. The other *cracked* specimens include two thoracic vertebrae (one of a small ungulate, one of a large ungulate) and a lumbar vertebra of a medium ungulate.
4. Fifty specimens (4.3% of the total NISP) are *crenulated*, resulting mainly from directed chewing on exposed bone surfaces. As expected, based on their (at least partial) flat morphology and thinly distributed cortical bone, most crenulation is preserved on axial and girdle elements, concentrated (by definition) on specimen terminations (Figure 5D; Figure 6A–C; Figure 6H). Only two LLB specimens–a cow femur (OB 7) and a red deer humerus (OB 6) –have *crenulated* ends, which are, in these cases, essentially the diaphysis-ward extension of intensive *furrowing* that commenced on trabeculae-filled epiphyses. Proper *furrowing* of LLB (excluding meatless metapodials) epiphyses (resulting from bear chewing that was directed in order to gain access to intrabone grease and red marrow) occurs on 17 of 64 (26.6%) of the recovered non-metapodial LLB specimens (Figure 7A–D). In contrast, only 2.9% (NISP = 19) of the total axial element NISP of 646 is *furrowed* (the basicranium of a goat [OB 12] and patella of red deer [OB 4] also show damage that is best characterized as *furrowing*). Together, humeri (NISP = 6) and femora (NISP = 6) account for 70.6% of the *furrowed* LLB sample, with all six humeri displaying *furrowing* on their proximal epiphyses only. For damaged femora, *furrowing* is distributed more evenly anatomically, with some femora *furrowed* only proximally, others *furrowed* only distally and still others on both epiphyses. Only one femur of goat (OB 12) shows a more intensive consumption on its proximal epiphysis, leading to an almost incipient *scooping-out*; however, the head is still preserved and this alteration therefore cannot be considered as a *classic scooping-out*. Four tibiae and one ulna are also *furrowed*.
5. Tooth *punctures*, *pits* and *scores*–the incidental results of bone defleshing–are fairly common in the aggregated bear-created sample, with *punctures/pits*-only affecting the most specimens (NISP = 98; 57.9% of the total NISP), *score*s-only on 45 specimens (26.6% of the total NISP), co-occurrence of both alterations on 26 bones (15.38% of the total NISP) and *punctures/pits*-plus-*scores* on 169 specimens (14.4% of the total NISP) (Figure 5E). One hundred thirty-three axial specimens and 14 girdle specimens are *punctured*, *pitted* and/or *scored* (Figure 6E–G). Very few skull specimens (NISP = 3) are *punctured*, *pitted* and/or *scored*. Likewise–and in conspicuous contrast to faunas modified by most other large carnivores (e.g., [6], [14], [15], [51], [58], [59], [61], [67], [72], [78], [88])–few of the recovered LLB specimens are *punctured*, *pitted* and/or *scored*. Moreover, none of the *punctured/pitted/scored* LLB specimens in the bear-derived sample is damaged on its diaphysis; instead all *puncturing/pitting/scoring* is restricted to LLB epiphyses. The complete lack of bear-induced diaphyseal *puncturing/pitting/scoring* also contrasts strongly with patterns observed in faunas modified by other major carnivoran taphonomic agents. These results are, however, consistent with Haynes [53]’s and Sala and Arsuaga [56]’s observations that bears do not puncture or pit LLB diaphysis, which have dense, thick cortices–but, instead, restrict their puncturing and pitting to areas of bone with thin layers of cortical bone encasing trabeculae (such as LLB epiphyses) or other thin cortical bone portions (such as scapula blades). In summary, the combined findings of our group, of Haynes [53] and of Sala and Arsuaga [56] contrast sharply with those of Saladié et al. [55], who recorded much more intensive puncturing, pitting and scoring of bone specimens, including heavy marking on bone portions with thick cortices, by captive bears.
  The size of *punctures/pits* produced by the Pyrenean brown bears ranged from <0.1×0.1 mm to 8.7×7.3 mm, with the majority of marks around 3.2–2.6×2.4–1.7 mm (Figure 8; Table S1; Figure S1). This range is consistent with that documented in other bear studies by Saladié et al. [55] and Sala and Arsuaga [56]. Small bears created the largest marks in our sample, including those on the remains of a horse (OB 1) and those on red deer bones (OB 15). The only other occurrence of bones that comes near to preserving similarly large marks was created by a large bear that fed on small roe deer. On the face of it, it seems counterintuitive that it was small bears who created the largest tooth marks in our aggregated sample. We suspect that small, young bears, recently on their own, might face more intraspecific competition for carcass resources and thus, when able, extract carcass nutrients more intensively and completely than larger, older bears.

### Modification patterns on LLB: Comparative results

**Figure 9:**
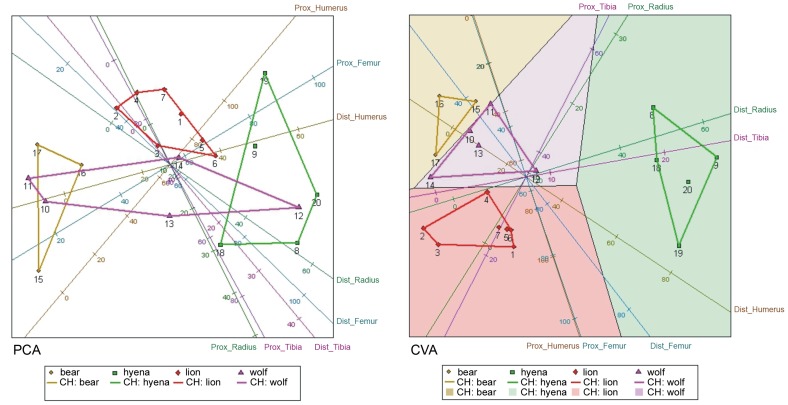
PCA and VCA plots comparing bone damage created by four carnivore species on ungulate long limb bones.

PCA yielded a two-dimensional solution accounting for 61.48% of the sample variance. The Eigen value for the first dimension is 45.13% (explaining most of the variance) and the second is 16.35%. Most variables display a similar value, with *proximal radius* and *proximal tibia* having the least impact on the final solution (Table 7). Considering both components, these portions, together with both femoral epiphyses, show a reduced impact compared to other LLB portions. Both ends of the humerus (especially the distal epiphysis) display a higher discriminatory value for differentiating taphonomic agents (Figure 9). This may have to do with taxon-specific styles by which different carnivores consume ungulate forelimbs.

**Table 7:** Matrices with the loading scores of the axis predictivities for principle component analyses (PCA) and canonical variate analyses (CVA).

	PCA		CVA	
	Dimension 1	Dimensions 1 and 2	Dimension 1	Dimensions 1 and 2
P. Humerus	0,603	0,911	0,331	0,991
D. Humerus	0,666	0,684	0,909	0,997
P. Radius	0,255	0,619	0,364	0,564
D. Radius	0,581	0,685	0,958	0,978
P. Femur	0,519	0,588	0,246	0,712
D. Femur	0,463	0,623	0,608	0,831
P. Tibia	0,296	0,659	0,502	0,912
D. Tibia	0,415	0,631	0,961	0,967

Abbreviations: P = proximal; D = distal.

CVA yields a two-dimensional solution that explains 69.5% of the sample variance, with the first dimension explaining 53.6% of that variance. This first dimension was mostly concerned with intergroup differences based on comparisons of the *distal humerus*, *distal radius* and *distal tibia*, and, to a lesser degree, the *distal femur*. In distinction, the proximal humeral, radial, tibial, and femoral epiphyses were substantially less useful for differentiating groups in the first dimension. The *proximal humerus* was the most useful discriminator in the second dimension. Thus, with regard to modification of ungulate humeri (especially the distal epiphyses of humeri), the CVA supports the PCA in stressing distinct patterns created by brown bears, spotted hyenas, lions and wolves–the four major taphonomic agents investigated here. In addition, the ungulate *distal radius* clearly separates the taphonomic impacts of the comparative carnivores in Euclidean space. In sum, the proximal and (especially) distal epiphyses of ungulate humeri and the distal epiphyses of ungulate radii are modified most intensively by spotted hyenas, next most intensively by wolves, and significantly less intensively by lions and brown bears. These comparative conclusions are apparent in the mean relative absolute error for each bone portion, with the *proximal* (PCA) and *distal* (CVA) *humerus* and the *distal radius* displaying the lowest values.

**Figure 10:**
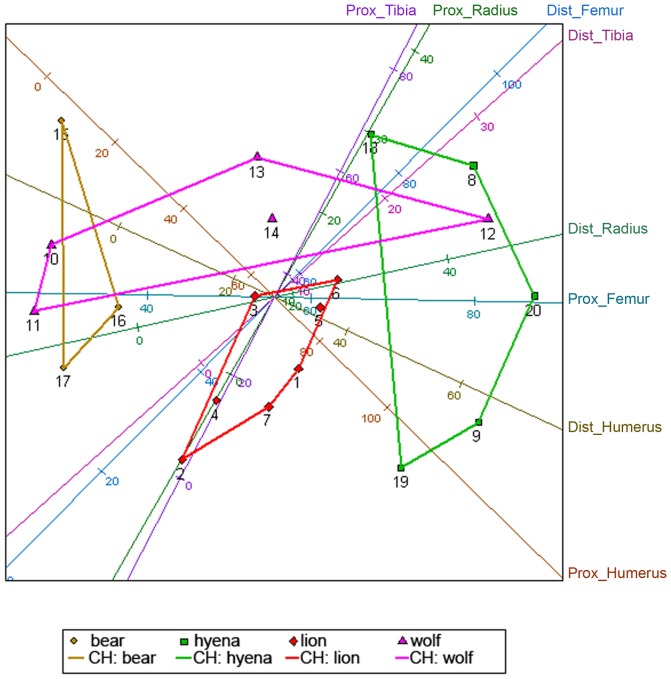
MDS plot showing a two-dimensional solution for four carnivore species and ungulate long limb bones.

Additionally, a two-dimensional, MDS-yielded solution shows that the proximal and distal epiphyes of humeri are the most influential first-dimensional variables (Figure 10). The *distal humerus* also had the highest score for the second dimension. The *distal radius* and *distal femur* were also highly influential in the first dimension (Table 8). These LLB portions also show the smallest mean relative absolute error (Table 9).

**Table 8:** Matrix of regression coefficients for the two-dimensional solution for the multidimensional scaling analyses.

	Dimension 1	Dimension 2
P. Humerus	0,421	−0,411
D. Humerus	0,404	−0,186
P. Radius	0,149	0,261
D. Radius	0,344	0,076
P. Femur	0,341	−0,006
D. Femur	0,281	0,28
P. Tibia	0,224	0,429
D. Tibia	0,169	0,151

Abbreviations: P = proximal; D = distal.

**Table 9:** Mean relative absolute error for the two-dimensional solution obtained for principle component analyses (PCA), canonical variate analyses (CVA), and multidimensional scaling analyses (MDS).

	PCA	CVA	MDS
P. Humerus	6,56	16,3	8,8
D. Humerus	15,7	7,1	12,8
P. Radius	14,4	18,4	15,3
D. Radius	13,7	11,8	15,7
P. Femur	13,7	18,1	17,8
D. Femur	16,1	23,5	14,6
P. Tibia	13,2	22,5	10,9
D. Tibia	14,8	17,5	15,9

Abbreviations: P = proximal; D = distal.

Collectively, the results of all three statistical analyses converge to show that the four major taphonomic agents investigated here are readily separated based on the relative intensity that each modifies LLB epiphyses. As predicted by the general, observationally based understanding that spotted hyenas and wolves are (in contrast to brown bears and lions) durophagus carnivorans, these two subjects generated the most intense taphonomic damage observed on LLB specimens. On the intrasample scale, the wolf-generated sample evinces the greatest variability of all four samples. We propose this high intrasample variability is, at least in part, a product of the aggregated nature of the wolf-generated sample, with some ungulate bones being those of young animals, modified over the course of several feeding/bone chewing episodes, while others derive from adult individuals [67]. However, we also note that the spotted hyena-generated sample is similarly variable to that generated by wolves; together, the considerable intrasample variability of the spotted hyena- and wolf-generated samples stands in contrast to the reduced intrasample variability of the brown bear- and lion-generated samples. This clear separation between the two groups–spotted hyenas and wolves on one hand, brown bears and lions on the other–probably reflects a more generalizable separation between durophagus and non-durophagus carnivores: the latter, acting primarily as carcass eviscerators and defleshers–and *not* as bone-crackers–avoid or minimize bringing their teeth in contact with bone surfaces; the former feed with less compunction–not only consuming innards and flesh but also readily demarrowing bones, and, in the course, inflicting a high degree of damage to LLBs. This broad contrast between durophagus and non-durophagus carnivorans is readily apparently in our PCA (and to a less extent in our CVA), which shows obvious separation between spotted hyenas and the other taphonomic agents that we investigated.

**Figure 11:**
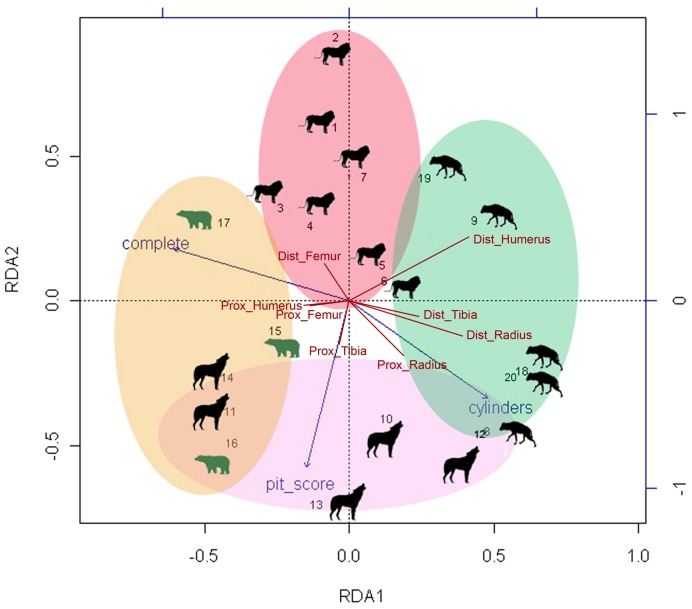
RDA analysis combining modifications on ungulate long limb bone ends according to carnivore species.

The RDA that combined the LLB epiphysis-modification dataset and the dataset composed of LLB completeness frequencies and tooth pit:tooth score ratios yielded a two-dimensional solution whose accumulated constrained eigenvalues (total explained variance) amounted to 96.9%. The first dimension explained 73.6% of the sample variance and the second dimension accounted for 23.3% of sample variance. The most influential variables for the first dimension are the *distal humerus*, *distal radius* and *distal tibia* (Table 10). The two most important constraining variables are LLB completeness frequencies, which explain most of the variance in the first dimension. The tooth pit:tooth score ratio accounts for a substantial part of the variance of the second dimension (Figure 11).

**Table 10:** Loading scores for each of the subordinate and constraining variables.

	RDA1	RDA2
P. Humerus	−0,11	−0,09
D. Humerus	0,42	0,22
P. Radius	0,18	−0,18
D. Radius	0,38	−0,11
P. Femur	−0,08	−0,01
D. Femur	−0,08	0,12
P. Tibia	−0,03	−0,14
D. Tibia	0,24	−0,05
Tooth pit: tooth score	−0,23	−0,88
LLB cylinders	0,74	−0,52
LLB complete bones	−0,94	0,28

Abbreviations: P = proximal; D = distal; LLB = long limb bone.

**Figure 12:**
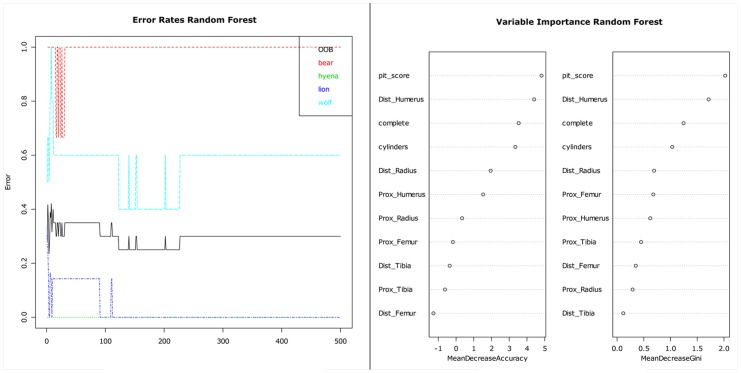
RF test combining ungulate long limb bone portions and the additional set of tooth pits to scores ratio and frequency of long limb bone cylinders and complete long limb bones.

The RF test that employed all variables produced a solution in which the OOB error was stabilized after 230 trees, although data on brown bear- and spotted hyena-induced modifications (representing, respectively both extremes of LLB modification) required fewer than 100 trees (Figure 12). The result produced an OOB estimate of the error rate of 30%. The solution is correct 70% of the time. The mean decreased Gini value showed that the most important variables were the tooth pit:tooth score ratio, *distal humerus*, LLB completeness frequencies, *distal radius* and *proximal femur*. Data for spotted hyenas and lions produced no error of classification, but, until the OOB was stabilized, data for wolves and brown bears were confounded up to 60% of the time (see also the proximity and overlap in PCA and CVA tests).

**Figure 13:**
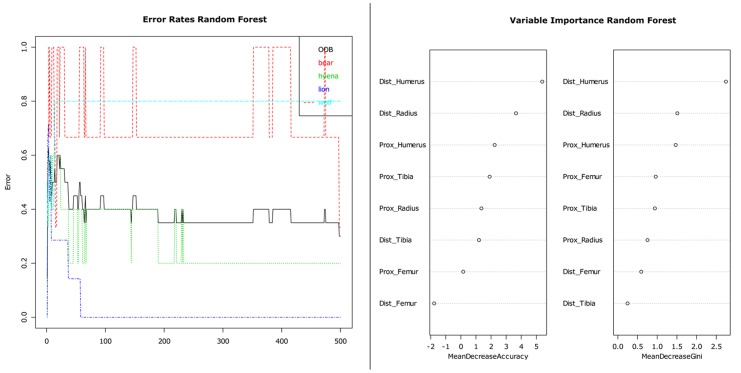
RF test combining ungulate long limb bone portions.

The RF analysis of LLB portion data only yielded a less stable OOB error (Figure 13), although it was similar to the previous analysis (30%). The mean decreased Gini value showed that the *distal humerus* and *distal radius* are the most discriminant variables, followed by the other portions. Brown bears signatures overlapped with those produced by wolves wolves >50% of the time until OOB stabilization was reached.

**Figure 14:**
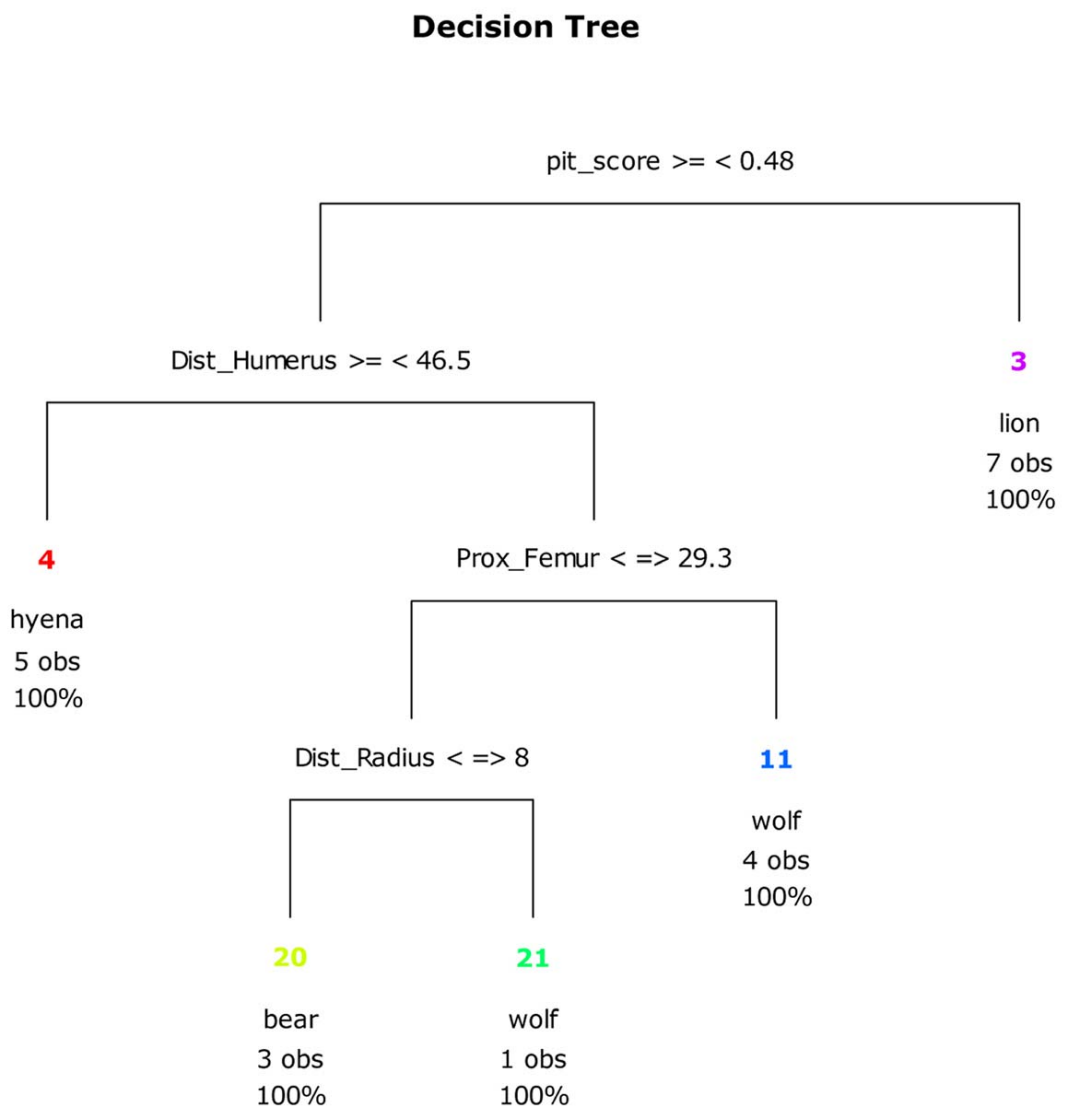
CT plot using ungulate long limb bone portions, the pits to scores ratio and the frequency of long limb bones cylinders and complete long limb bones.

A CT with the selected set of variables from the first RF test shows that tooth pit:tooth score ratios of <0.48 correctly identify lion-generated samples 100% of the time (Figure 14). If modifications on the distal humerus are >46.5%, this correctly classifies hyenas in 100% of the observations. If the damage on the distal humerus is <46.5%, then only a combination of damage on the proximal femur and the distal radius can correctly differentiate bears from wolves in 100% of the observations.

**Figure 15:**
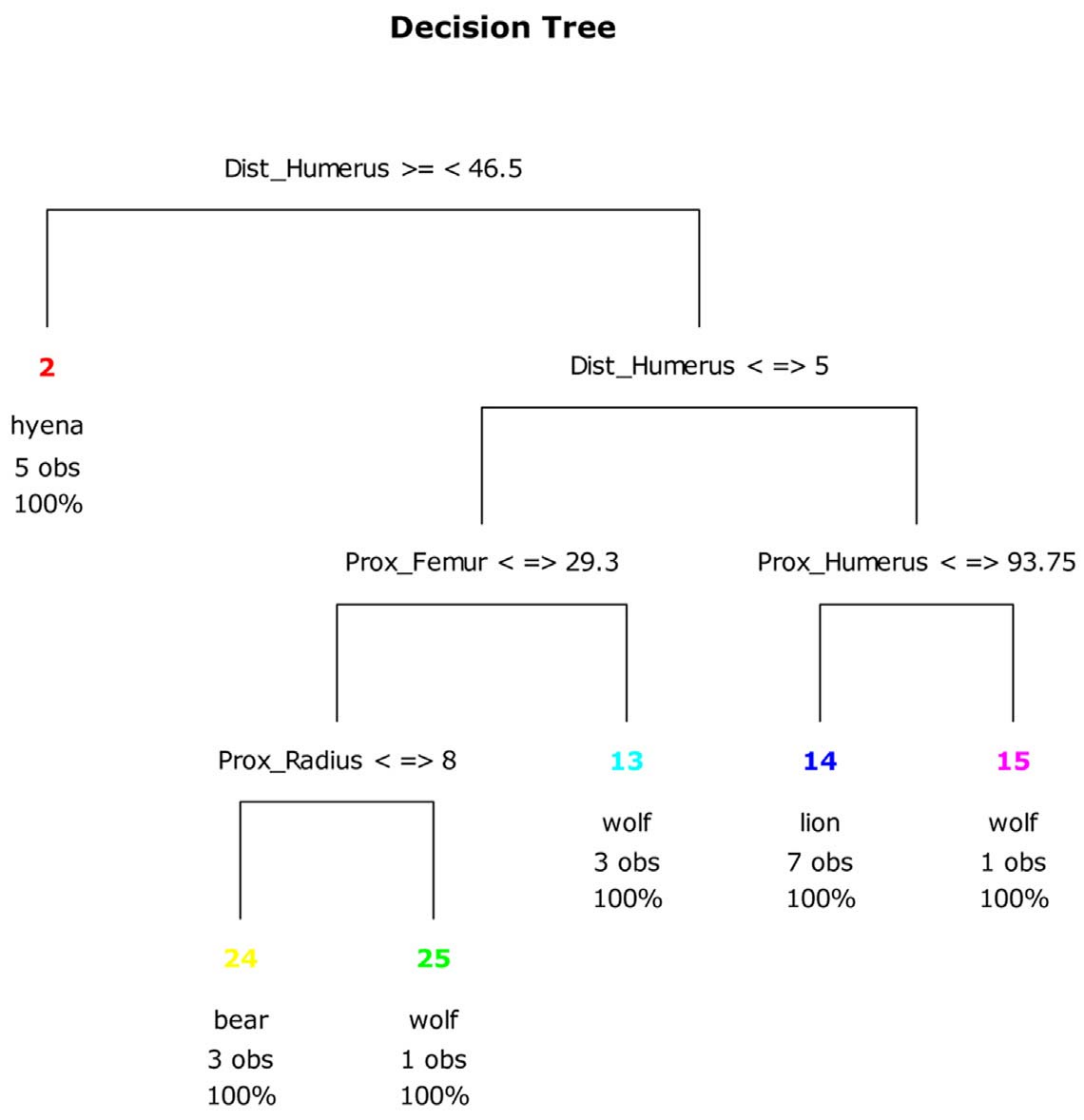
CT plot using only ungulate long limb bone portion variables.

Further, our CT analysis of LLB portion variables reveals that modification distributions of the *distal humerus* differentiate spotted hyena-derived samples from those produced by the other three taphonomic agents that we investigated. Last, consideration of damage frequencies on the *distal humerus*, *proximal radius* and *proximal femur*, in combination, differentiates samples modified by brown bears wolves in 100% of the observations (Figure 15).

## Conclusions

Previous neotaphonomic studies demonstrated that bears are efficient consumers of small-sized ungulate carcasses, resulting taphonomically in modest degrees of bone damage and a lack of medium- or long-distance transport of carcasses from points of initial discovery [36], [53], [55], [56]. These results, along with those on the morphology, metrics and anatomical distribution of their tooth marks, prompted some workers (e.g., [55]) to characterize the taphonomic potential of bears as consistent with that of better-studied lions. Data and results presented here disagree with this assessment. Most of the bear-imparted damage in our sample occurs not on LLB specimens, but instead on axial and girdle specimens.

That said, compared to previous neotaphonomic studies of bears (e.g., [53], [55], [56]), the Pyrenean brown bears *did* inflict a relatively high frequency of tooth punctures/pits/scores –on 14.4% of the total aggregated NISP. Consistent with the results presented by Haynes [53] in a different sample of bear-modified bones, most of the pits and scores in our sample are light and are positioned on thin cortical bone; there is a very modest degree of furrowing into the trabeculae of LLB epiphyses, but no substantial punctures through the thick cortices of LLB diaphyses (the only perforated bone in the Pyrenean bear-modified sample is a red deer rib [OB 15]). Based on the complementary results of these independent studies of bears, we feel comfortable generalizing that bears–unlike spotted hyenas and other durophagus carnivores–do not regularly use their teeth to puncture LLB shafts. Following from this conclusion, we remain cautious about well-known interpretations that fossil LLB diaphyseal specimens from the Middle Pleistocene cave sites of Yarimburgaz (Turkey) [31]–[33], Troskaeta (Spain) [36], [89] and Divje Babe (Slovenia) [54] were punctured by extinct cave bears during the course of cannibalistic feeding. Although it is possible that bears at these sites fed with great intensity (perhaps under stressful environmental and/or individual conditions; see, e.g., our discussion of OB 15, above) and did, in fact, impart punctures on LLB diaphysis, we believe that, additional detailed analyses are required, since the punctured bear LLB diaphyses may have also been damaged by the taphonomic actions of durophagus carnivores with robust, conical tooth cusps, such as hyenas or wolves. Indeed, by refining our understanding of the taphonomic behaviors and consequences of extant brown bears, this study, in stepwise manner, contributes to ongoing efforts to model the possible impact of extinct ursids on conditioning paleontological and archaeological faunas in (especially) European Pleistocene contexts.

More generally, the results of our comparative statistical analyses draw the most precise distinctions between damage patterns imparted respectively by durophagus and non-durophagus carnivorans. By in large, these results corroborate commonsense and earlier qualitative observations that durophagus carnivorans can inflict much more intensive damage to bone and are more effective bone destroyers than are non-durophagus carnivorans. But the novel contribution of this research is that it provides quantitatively based, taphonomic thresholds for several commonly preserved paleontological variables–thresholds that can be used, with great accuracy and nearly 100% confidence, to separate faunas formed predominantly by durophagus carnivorans from those formed predominantly by non-durophagus carnivorans.

In summary, the observations and data that emerged from this study provide a clear pattern of ungulate carcass consumption by Pyrenean brown bears, regardless of bear age, sex or body size. But, considering the impact of local environment on conditioning taphonomic behavior (e.g., [14]), additional investigations of extant ursids in other locales and habitats is certainly merited. Ultimately, results from all these studies should be combined in order more completely characterize the taphonomic potential of extant brown bears and to better define their role as referents for modelling the taphonomic behaviors of their extinct cogeners.

## Supporting Information

Figure S1 Dimensions of bear tooth pits and punctures occurring on cancellous bone and on thin cortical bone, plotted by size of ungulate bones (A1–A3) and size of bear consumers (B1–B3). OB = Observation.
(TIF)

Table S1 Descriptive statistics for length and breadth (*n* =  number of cases, CI =  Confidence Interval [±95%], SD =  Standard Deviation, CV =  coefficient variation) of bear tooth pits, punctures and scores. Length and breadth are shown in millimeters.
(XLS)

Video S1 Small bear consuming the carcass of a red deer.
(MP4)

Video S2 Large bear with the carcass of a cow.
(MP4)

Video S3 Bear fracturing a deer spine in order to access internal edible soft tissues.
(MP4)
