# Supplementary figures and images for: The “Bear” Essentials: Actualistic Research on Ursus arctos arctos in the Spanish Pyrenees and Its Implications for Paleontology and Archaeology
Source: PLoS One. 2014 Jul 16;9(7):e102457. doi: 10.1371/journal.pone.0102457 (PMC4100921; doi:10.1371/journal.pone.0102457)

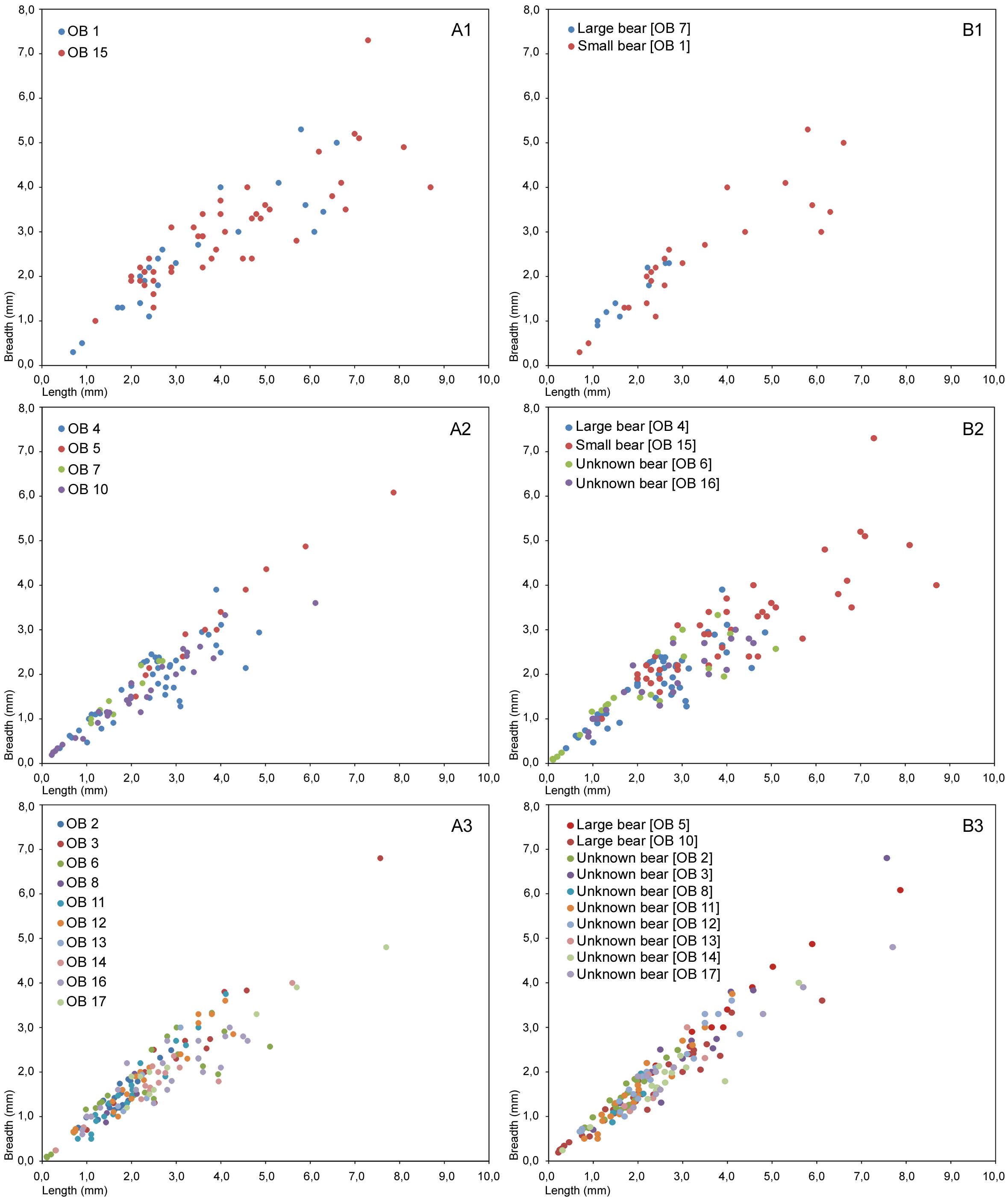

Supplement: Figure S1 — Dimensions of bear tooth pits and punctures occurring on cancellous bone and on thin cortical bone, plotted by size of ungulate bones (A1–A3) and size of bear consumers (B1–B3). OB = Observation. (TIF) [file pone.0102457.s001.tif]
